# Shifting Values and Shifting Risks: Debates on the New Biology in Germany and the United States Before and After Asilomar (1960–1980)

**DOI:** 10.1007/s10739-025-09831-w

**Published:** 2025-11-17

**Authors:** Christina Brandt

**Affiliations:** Friedrich Schiller University Jena, Ernst-Haeckel-Haus, Jena, Germany

## Abstract

This article argues that while the 1975 Asilomar Conference on Recombinant Molecules gave rise to new developments in science policy, the subsequent debates about genetic engineering should not be viewed in isolation, but rather in the larger historical context of discussions on a *new biology* that began as early as the 1960s. The new field of molecular biology was at that time already hotly debated by holistic biologists, sociologists, and philosophers in West Germany. In part, these debates were a reaction against the utopian views of some leading US scientists whose imaginaries about cloning and genetically modified man came under serious critique after the CIBA Foundation symposium *Man and his Future* (1962). As a result, some German philosophers, scientists and sociologists framed the new biology against the historical background of Nazi eugenics. In the 1970s and 1980s, when the new social movements (peace movement, anti-nuclear power movement, ecology movement, feminist movement) started to debate genetic engineering, the public and political controversies about recombinant DNA technologies remained closely intertwined with issues of cloning, embryo research and in-vitro fertilization (IVF). This discursive assemblage was often framed by dystopian fears about human genetic engineering. The article traces these multi-layered discourses and analyzes the continuities and changes in the debates from the 1960s to the 1980s in West Germany while comparing them with developments in the United States. It demonstrates how different understandings of gene technologies and their future impact on society collided and how different concepts of risk developed among both scientists and the public after Asilomar.

## Introduction: Asilomar and Debates on Gene Technologies in Historical Context

Whether genetic research will be a curse or a blessing depends not only on the pace of development, but much more on the goals. These goals can, at best, be only roughly recognized, since we are only at the beginning of the *genetic era (Genzeitalter)*. Therefore the question: ‘Genetic research – a curse or a blessing?’ cannot be answered as definitively as it was posed. After reading this volume, however, there can be little doubt that the *essence of the human being has never been so threatened by genetic engineering as it is today.* (Flöhl 1985, p. 375, translation and italics C.B.)^1^

The existential threat to humans posed by the newly emerging genetic era was a widespread topic of (West) German public discourse in the mid-1980s. It was with this gloomy prognosis that Rainer Flöhl, science editor of the *Frankfurter Allgemeine Zeitung* (FAZ), concluded the summary of a collective volume he had published under the title: *Genforschung—Fluch oder Segen?* (Genetic Research: Curse or Blessing?) in 1985. Flöhl, who held a doctorate in chemistry, was well versed in debates on genetic research. As a science journalist and head of the FAZ’s *Nature and Science* section, he was an early and influential voice in the journalistic coverage of genetic engineering in West Germany. In 1975, he was the only German journalist to attend the *International Conference on Recombinant DNA Molecules* in Asilomar, California,^2^ where some 140 scientists discussed the potential safety risks of this new field of research, largely behind closed doors. By the time Flöhl published the aforementioned book ten years later, the public debate in West Germany had reached an apex, and issues of gene technologies had become a hotbed of controversy in science policy, with far more at stake than just problems of laboratory safety or questions about a research moratorium on specific experiments. Particularly noteworthy is the choice of words: the title of Flöhl’s edited volume framed genetic research in moralistic terms as a “curse or blessing” (Flöhl 1985, p. 375). As most of the articles by prominent philosophers, theologians and some scientists in the anthology made clear, German discourse on genetic research was highly normative. The *politicization* and *moralization* of the new life sciences overlapped in the West German controversies in the 1980s, where these debates had a degree of intensity that far exceeded similar discussions in France, Great Britain and the United States.

This article analyzes these debates on genetic engineering and the politics of science in West Germany and the United States in the broader context of scientific and cultural developments from the 1960s to the early 1980s. According to a standard narrative, public debates about genetic engineering began with the advent of recombinant DNA technologies in the early 1970s, which has often been taken to mean not earlier. Historians of science have often seen the conference on recombinant DNA research in Asilomar in 1975 as a symbolic starting point of a rapidly developing, heated public debate on issues of laboratory safety and public health hazards. After 1972-1973, when two research groups in California conducted the first genetic engineering experiments on *E. coli* (Jackson et al. 1972; Cohen et al. 1973), this work raised questions about safety, and a research moratorium for specific experiments was demanded by some scientists themselves, most prominently Paul Berg, Maxine Singer, and others (Berg et al. 1974; Singer and Söll 1973). These events crested in the 1975 Asilomar conference and its call for the creation of guidelines for safe practices for the new field of recombinant DNA research, but also for a public movement critical of genetic engineering in the years that followed, particularly in the United States.^3^ Post-Asilomar debates ultimately spurred regulations and guidelines for scientific research in Western countries, though they varied from country to country (Jasanoff 2005; Gottweis 1998; Wright 1994).

However, while it is certainly true that *political* issues of science regulation took on a new dynamic in the 1970s, public discussion of the future impact of the life sciences on people and society can be traced back much further. Thus, this article does not treat Asilomar as the starting point of an entirely new discussion about the risks of genetic engineering, but rather situates the Asilomar conference within a larger historical context, treating it as both a turning point but also as just one moment in a longstanding public debate about the new life sciences. This change of perspective is all the more important when it comes to understanding the specific situation in Germany and the question as to why controversies over genetic engineering as a new biopolitical era of genetic modifications of humans became so heated in the 1980s. Scholars have often maintained that the United States was the “country of origin of the genetic engineering debate” in the 1970s (Wieland 2012, p. 71), and that the debate on genetic engineering was slow to unfold in West Germany. The dominant perspective is thus that the discussion started late in Germany, but then took on an incomparably more intense dynamic (Salem 2013; Jasanoff 2005). The explosive nature of the German debates in the 1980s, at times marked by strong emotions, has often been described as a distinctively German phenomenon. Sheila Jasanoff for example speaks of “programmatic fears” (Jasanoff 2005, p. 58). She delineates the features of West German discourse, writing: “Public debate about biotechnology was slower to gain momentum in Germany than in the United States (and slower still in Britain), but when it did, it drew on a particular reservoir of national memories, experiences, and political activism with no close analogues in any other Western country” (Jasanoff 2005, p. 58).

However, I think we should question some aspects of this historical standard narrative. While it is certainly true that the debates in West Germany were much more controversial than in other Western countries, we should approach the notion of a delayed debate with some skepticism. Those who claim that the German debates started with a delay tend to base their arguments on a comparison with the post-Asilomar situation in the United States. They also tend to narrow their focus to debates on *risks* of recombinant DNA research for individuals, society, or ecology — and related *political* controversies about science regulation. However, there was not just one debate, but many, and in the public sphere the uniquely German term *Gentechnologie* (gene technology) — as well as the notion of *genetic engineering*, widely used in the United States — became catch phrases for diverse developments in the life sciences. As this article will show, the situation was highly complex, with overlapping debates on different issues. Especially in Germany during the late 1970s and well into the 1980s, the public imagination mingled recombinant DNA research, reproductive medicine, embryo research, and cloning research to such an extent that many molecular biologists (who saw the recombination of DNA in simple organisms as strictly separated from genetic research on humans) vehemently criticized public debate for completely distorting the genetic engineering issue. The West German public debates on gene technologies were thus a kind of discursive *assemblage* that fused issues that many molecular biologists did not feel belonged together, or at least not in this form. This complex discourse engaged not only with the political regulation of the *risks* of genetic engineering, but, more fundamentally, also with *values* or normative questions about the domination of science and technology over nature.

At the heart of these public discourses was the idea that humanity was witnessing the dawn of a new era of genetic engineering. The perception of genetic technologies as a threat to the “essence of the human being” and related dystopian imaginations of a new era of biotechnological human manipulation remained central motifs in the highly normative German debates in the 1980s (Flöhl 1985, p. 375), having begun two decades earlier. Understanding the discursive dynamics in the decade after Asilomar necessitates that we look to the 1960s, when controversies about what was called a new biology and the future of humanity were already taking place *in both countries.* The *political* regulation debate that began in the 1970s was preceded by *moral* debates in the 1960s about the future impact that modern life sciences might have on humans and society. Thus, the landmark conference in Asilomar 1975 narrowed down these previous discussions about a *biological revolution* dating back to the 1960s, when, according to Nathan Crowe, cloning and the “transformative powers of the biological sciences” for society were widely discussed and played an important role for the foundation of bioethics in the United States (Crowe 2021, pp. 12–14).

The first section of this article turns to this history and examines more closely how the discourse on a *new biology* and its future implications for humans and society emerged, particularly in the United States in the 1960s, a decade characterized by broad engagement with modernity and the future. I argue that the emerging idea of a “biological revolution” (Crowe 2021, p. 12) and related utopian views of future cloning and manipulative power to alter human nature and reproduction should be understood historically in the context of older eugenic visions of optimization that were carried into the age of molecular biology. The key contributions that two scientists made to this new utopian discourse—Hermann J. Muller and Joshua Lederberg—are discussed in more detail. Muller (1890-1967) and Lederberg (1925-2008) in particular were the subjects of vehement criticism in German debates in the second half of the 1960s. The third and fourth part of the article then explains how the approaches of the new biology or biological revolution were received critically in West Germany, where, by the mid-1960s, an intensive preoccupation with questions of the future had taken hold. In particular, the utopian, sometimes far-reaching ideas about human cloning and a genetically modifiable future mankind so widely (and sometimes enthusiastically) discussed by scientists in the United States were regarded as a scientific dystopia. Holistic biologists, sociologists, philosophers, and others in West Germany in the 1960s criticized what they saw as these ideas’ continuities with the biopolitics and eugenics of National Socialism and thus strictly rejected them. The fifth and sixth parts are concerned with the 1970s, often portrayed as a decade of multiple crises. A new generation of molecular biologists shaped the scientific discourse on recombinant DNA research, but the debates of the 1960s continued to reverberate in the public sphere. From a comparative perspective on the United States and West Germany, it is shown that the scientific policy debate that emerged with the Asilomar conference on laboratory risks, security issues and research regulations has narrowed the focus of the debates, but that controversial ideas of “human genetic engineering”^4^ nevertheless remained highly virulent, especially in the public perception. The last part discusses how this reverberation affected the perception of genetic utopias. While in the 1960s it was scientists themselves who propagated science fiction-like visions of the future involving genetically enhanced humans, in the risk debates of the late 1970s and early 1980s molecular biologists vehemently criticized this blurring of the boundaries between fact and fiction as speculative and imaginary when, ironically, concerned members of the public repeated what scientists had said earlier.

## The New Biology and the Future of Man: An Emerging Discourse in the United States in the 1960s

The first publicly recognized successes of molecular biology—in particular the “cracking”^5^ of the genetic code between 1961 and 1966—and the rapid institutionalization of this still young discipline increasingly fostered the perception of modern biology as the new leading science of the future (Kay 2000). In addition to the emergence of molecular biology, experimental findings from cloning research—somatic cell nuclear transplantation in embryology and developmental biology—gave rise to far-reaching ideas about the future of mankind. Nathan Crowe and others have detailed how cell nuclear transplantation experiments with frogs in the 1950s and 1960s (Briggs and King 1952; King and Briggs 1956; Gurdon et al. 1958; Gurdon 1962), which themselves did *not* aim at the reproduction of identical organisms, were soon received by journalists, novelists and bioethicists as suggesting the possibility of cloning humans (Crowe 2021, 2014; Brandt 2009a, 2009b, 2007; Maienschein 2003). These imaginaries of future human cloning became widespread in the second half of the 1960s and the early 1970s. They contributed to the then widespread view that a “biological revolution” was underway (Crowe 2021, pp.163–231). When these cloning scenarios began to take shape, however, they were often linked to older eugenic visions of improving the so-called human race by controlling human reproduction. The discussion of a “revolutionary new biology” that began around 1960 grew almost seamlessly on these older discourses in its utopian underpinnings (Sonneborn 1965, p. 1). This is partly due to the fact that those scientists Daniel Kevles has identified as representatives of reform eugenics (Kevles 1995, p. 173)—Hermann J. Muller, J. B. S. Haldane and Julian Huxley—were strong voices in the revived debate. Geneticist Muller played a most prominent role in the popularization of the idea of human cloning in the 1960s and propagated it, in line with his eugenic visions, as a form of reproduction for the future (Brandt 2009a, b).

The impact of the new biology on the future of man and society was already a major theme around 1960. This is illustrated by a number of prominent conferences that took place in the United States and the United Kingdom in the late 1950s and early 1960s. Their titles even referred explicitly to the question of humanity’s future in the context of new developments in science and technology. The CIBA Foundation Symposium on *Man and His Future* that took place in London in 1962 is probably the best known of them. There, prominent scientists discussed the consequences of the “biological revolution” (Jungk and Mundt 1966) for society. However, it was by no means the only conference. Shortly thereafter, in April 1963, geneticists organized a conference on *Prospects for the Experimental Control of Human Heredity and Evolution* at the Ohio Wesleyan University (Sonneborn 1965). Already in 1957, the Seagram Company, a leading manufacturer of drinks, celebrated a centennial with the theme *The Next Hundred Years*. This conference was followed by the inauguration of the iconic Seagram skyscraper in New York in 1959 with an event on *The Future of Man*. The media in the United States published widely about the Seagram events, where leading scientists, philosophers, novelists and public intellectuals gave talks about their views of the future (Frost et al. 1959).

Muller, who had been awarded the Nobel Prize in Physiology or Medicine 1946 for his leading research in radiation genetics, was one of the keynote speakers at almost all of these events. His ideas were thus widely disseminated. The *New York Times*, for example, reported extensively about the 1957 Seagram symposium and published short summaries of the talks.^6^ Muller’s piece in the *New York Times* depicted a future world with “predictable offspring.” In the future, he predicted, a “more ethical attitude concerning reproduction” would be established, and it would “be regarded as a social obligation to bring into the world human beings as favorably equipped by nature as possible, rather than those who simply mirror their parents peculiarities and weakness.” While “offspring now ordinarily have their hereditary material picked in a random way from two different parents,” in the future there would be cases “where the offspring would obtain his hereditary equipment entirely from one individual with whom he is as genetically identical as if he were an identical twin.” A way of achieving this would be by means of somatic cell nuclear transfer, as Muller pointed out: “This will be accomplished, as is now done, by extracting the nucleus from a human egg and inserting in its place an entire nucleus obtained from a cell of some pre-existing persons, chosen on the evidence of the life he or she had led, and his or her drive potentialities.”^7^

Like his contemporaries Julian Huxley and J. B. S. Haldane, Muller belonged to a generation strongly influenced by the eugenics discourses of the early 20th century. Indeed, all three were themselves prominent proponents of eugenics.^8^ It is no coincidence that Muller introduced the idea of cloning by means of somatic cell nuclear transfer to the public in the late 1950s. As early as the 1930s, he had envisioned a scientific utopia focused on the control of reproduction and the decoupling of reproduction from the human organism (Muller 1935). In the following years and decades, Muller promoted the idea of artificial insemination (using sperm from what he saw as superior personalities) as a key eugenic technology of the future.^9^ For him, cloning by means of somatic cell nuclear transfer was not a categorically new step, but merely a further development of what he called “germinal choice” (Muller 1963, p. 247). At both the Seagram symposium in 1957 (which addressed a broad public) and in a 1959 lecture at the Darwin Centennial at the University of Chicago (which addressed a scientific audience) Muller discussed cloning as a future means of eugenically selecting human genotypes. His vision of the future went far beyond the factual knowledge available at the time.^10^ However, Muller referred to the somatic cell nuclear transplantation techniques developed by Briggs and King not explicitly as cloning, but as a “promising type of parthenogenesis” (Muller 1960, p. 453). Muller believed that the “high order of predictability” of this kind of parthenogenesis would represent a boon for society (Muller 1960, p. 454). In his talk at the Darwin Centenary, he described the creation of supposedly identical genetic copies of selected individuals, a scenario that became widespread in science fiction depictions of cloning in the following decades:

This type of parthenogenesis would make possible the production of multiple progeny that resemble their genetic parent and one another about as closely, phenotypically, as identical twins reared apart resemble one another and that are about as alike genetically as identical twins. Here, then, there would be an extremely high order of predictability regarding the nature of the children and virtually no regression except that occasioned by environmental factors, such as the manner of bringing up the children. Of course, the high general intelligence of the progenitor would in most cases be capable of being applied in very diverse directions in his separate manifestations, as we might term then. [ ...] When one considers how much the world owes to single individuals of the order of capability of an Einstein, Pasteur, Descartes, Leonardo, or Lincoln, it becomes evident how vastly society would be enriched if they were to be manifolded. Moreover, those who repeatedly proved their worth would surely be called upon to reappear age after age until the population in general had caught up with them. (Muller 1960, p. 454)

Muller not only discussed cloning, but also the direct modification of the human genetic constitution as a further technique for improving genetic disposition, for example by modifying nucleotide sequences of individual genes. By the late 1950s, speculation about this form of genetic engineering was in the air, which again was far ahead of what was actually known at the time. However, Muller was skeptical that this method, which he called “genetic surgery” (Muller 1965, p. 104) could be successfully developed in the future.^11^

It is well known that Muller was a lifelong eugenicist (Paul 1998, 1984; Carlson 1995, 1987; Meloni 2016, pp. 124–129). What is less well known, however, is how obsessed he became in the early 1960s with the idea of establishing a sperm bank that would hold the genetic endowment of Nobel Prize winners or other eminent men of science. In the several talks and articles he published in the late 1950s and early 1960s, he repeatedly and passionately expounded upon the same topic: the “means and aims in human genetic betterment” (Muller 1965, p. 100), and how artificial insemination and germinal choice could be developed as reproductive technologies of the future (Muller 1961, 1963, 1966). Muller’s efforts found public resonance in the United States, but they were also subject to strong criticism. The idea of germinal choice was seen as a threat to the traditional concept of sexual morality. The underlying reductionist view of human nature and the idea of the infinite perfectibility of human kind were both heavily criticized. However, the question of who would have the authority to decide on germinal choice in the future was also raised. Comparisons were often drawn with Aldous Huxley's *Brave New World*, and in religious circles, interfering with creation was strongly condemned as hubris, accompanied by accusations of playing God. In June 1962, the cover of an issue of *Sexology* (a New York-based magazine devoted to sex education that was founded by the science fiction writer Hugo Gernsback in 1933) read “Nobel Prize Winner Hermann J. Muller on Sperm Bank.”^12^ In a text with the title “Improving Man’s Genes: A Nobel Prize Winner Discusses His Sperm Bank Proposal,”^13^ Muller defended his ideas against public criticism. Not only was Muller's idea of germinal choice widely discussed in the print media, but also on television. In February 1960, *Close-Up*, a Canadian television show, broadcast a two-part report. The first week the playwright and novelist Arthur Hailey interviewed Muller. A week later the scientist Wendell Stanley, the Jesuit anthropologist Franklin Ewing, and professor of law Frank Scott discussed Muller’s statements critically. The interview with Muller concluded: “That was *Close-Up* glimpse of the future. A real future? And how far away? Will we ever place orders for babies the way we place orders for cars? Some scientists think we not only will but must, if the race is to survive. Does this mean challenging the rights of God Himself?”^14^

Joshua Lederberg was another highly influential scientist in the 1960s. More than any other molecular biologist at the time, Lederberg promoted the notion that the new biology could usher in a revolution for both the individual and society (Crowe 2021, pp. 169–194). A zoologist by training, Lederberg belonged to a new generation of molecular biologists. In 1958, at the age of 33, he was awarded the Nobel Prize (together with Georg Beadle and his former teacher Edward Tatum) for pioneering work in bacterial genetics that he had conducted with his former wife Esther Lederberg in the late 1940s and early 1950s. Although Muller and Lederberg belonged to different generations, there were strong parallels between their efforts to sensitize people to the future social changes that they believed would inevitably result from scientific progress. Both saw themselves as playing a similar role in this regard. When 41-year-old Lederberg began writing his series *Science and Man* for the *Washington Post* (see below) in 1966, 76-year-old Muller wrote appreciatively to Lederberg:

It is a remarkable series that has in my opinion out-Haldaned Haldane himself, being on the whole even sounder, partly because not so smart-Alecky. I hope it sets the beginning of a new kind of scientific writing for the public and the popular press. You could, though, jazz it up a little, to the advantage of its going-over and getting-to-feel-the-public aspect.^15^

And Lederberg responded:

You could not have made a more flattering allusion than your reference to Haldane, whose flair for commentary made an overt model for what I have been after too. Then, besides yourself and perhaps Huxley, who else is usefully putting himself out this way?^16^

Lederberg contrasted the traditional form of eugenics, which was based on an evolutionary, long-term model of selection, with what he called *euphenics*. He regarded “euphenics,” which was based on “developmental engineering techniques” (Lederberg 1963, p. 428), as a much more effective, faster means of controlling human individual development (see also Lederberg 1966a, p. 293). When introducing the new term euphenics in 1962-1963, Lederberg was inspired by Aldous Huxley. Shortly after the CIBA Foundation symposium on *Man and his Future*, which took place in November 1962, Lederberg sent the paper he had presented in London to the novelist in January 1963. Calling himself a “life-long admirer,” Lederberg emphasized that he could not have written “this paper without thinking how much more effective your own work has been and might be—and I hope you will recognize and accept the acknowledgements that are implicit in it.”^17^

In particular, Lederberg saw molecular genetics (and its future possibilities for controlling the transmission of genetic information) as well as cell biology as the two main fields that would enable scientific intervention in human nature. Particularly influential was an article titled “Experimental Genetics and Human Evolution” that Lederberg published in 1966 in slightly modified form in two prominent journals: in the 100th anniversary issue of *The American Naturalist*, the journal of the American Society of Naturalists (Lederberg 1966c), and in the *Bulletin of the Atomic Scientists* (Lederberg 1966b), a journal devoted to the relationship between science and society. A German translation was soon conducted at the Institute of Human Genetics of the University of Freiburg and was published only one year later (Lederberg 1967). In these texts, Lederberg developed a specific vision of “genetic design” (Lederberg 1966b, p. 9) that would be studied by a field he called *algeny*. By this he meant a kind of “genetic alchemy” (Lederberg 1966b, p. 11), a genetic transformation of the human being. Alternating between the roles of an admonisher and committed advocate, Lederberg primarily supported his arguments (similar to what was already evident in the utopian views of Muller) with his belief in the *inevitability* of a future shaped and guided by new biological technologies: “Recent discussions of controlled human evolution have focussed on two techniques: selective breeding (eugenics), and genetic alchemy (algeny). The implementation will doubtless proceed even without an adequate basis of understanding of human values, not to mention vast gaps in human genetics” (Lederberg 1966c, p. 530). For Lederberg, cloning and genetic modification were the two central directives of human beings’ future capacities of self-design:

Algeny presupposes a number of scientific advances that have yet to be perfected; and its immediate application to human biology is, probably unrealistically, discounted as purely spectulative (*sic*). In this paper, I infer that the path to algeny already opens up two major diversions of human evolution: clonal reproduction, and introgression of genetic material from other species. Indeed, the essential features of these techniques have already been demonstrated in vertebrates, namely, nuclear transplantation in amphibia, and somatic hybridization of a variety of cells in culture, including human. (Lederberg 1966c, p. 531)

Lederberg’s influence on the new biology debate can hardly be overestimated. Not only was he a key player in a number of important science policy committees in the United States from the mid-1960s onwards, but he also became known to a wide audience through the weekly columns he wrote for the *Washington Post* from 1966 until 1971. In this *Science and Man* series, he addressed key issues in medicine, life sciences, and technology, and discussed their implications for society. A particular focus of the series was developments in the life sciences that affect human reproduction. Lederberg’s short text “Unpredictable Variety Still Rules Human Reproduction,” in which he described future cloning of man as an “interesting exercise in social science fiction,” sparked a discussion on bioethics with molecular biologist-turned-ethicist Leon Kass. In response to the column, Kass wrote a harsh letter to the editor of the *Washington Post*, voicing his strong theological, moral and political objections to altering human reproduction. He argued that “programed reproduction of man will, in fact, de-humanize him.”^18^

Perhaps unsurprisingly, these futuristic eugenic ideas and their optimistic embrace of technology quickly found an echo in the popular sphere (Crowe 2021, pp. 208–214; Brandt 2009a, 2009b). In his 1970 bestseller *The Future Shock*, futurologist Alvin Toffler quoted an interview with Lederberg in which the latter stressed that cloning would “definitely happen within the next fifteen years” (Toffler 1971 [1970], p. 156). The British science journalist Gordon Rattray Taylor had already devoted an entire chapter to cloning in his 1968 book *The Biological Time Bomb*, a book that also spent months on German bestseller lists (Taylor 1969, pp. 25–36). But it was not only in the genre of popular science writing that cloning became a central theme in the late 1960s. Fantasies about cloning and outer space were also taken seriously and used as thought experiments for reflections on ethics. In 1972, the theologian and bioethicist Joseph Fletcher argued that situations might arise in the future in which cloning or positive eugenics would be justified. This was in response to Leon Kass, who had formulated some of the first ethical statements against cloning in the United States (see Kass 1972). Fletcher discussed Lederberg’s (1966a, 1996b, 1996c) proposition, undergirded with utilitarian logic, that clones might be deployed in outer space in the future.^19^

Future scenarios of cloning and human genetic modification were also seriously discussed in the academic world. In 1966, the Committee on Science and Public Policy of the National Academy of Sciences initiated a panel on *Biology and the Future of Man* in order to “state and define the most pressing problems which seem most promising for attack in the next several years, and to indicate the nature of current barriers to progress in this area.”^20^ The panel included (among others) geneticist Curt Stern, Lederberg, and geneticist and evolutionary biologist Theodosius Dobzhansky (who had devoted an entire chapter on “Muller’s Bravest New World” already in his 1962 book on *Mankind Evolving* (Dobzhansky 1966 [1962], p. 327). The group submitted its 30-page report to the National Academy of Sciences in 1968.^21^ It dealt mainly with questions of biology and medicine, genetic diseases, environmental pollution and demographic issues such as overpopulation. It also focused on the future of human reproduction and selection. Under the heading “Guarding the Genetic Quality of Man,” the report unabashedly asserted that “eugenic thinking should be encouraged notwithstanding tragic errors of the past.”^22^ While noting that the prospects for “genetic surgery” (understood as the replacement of “specific undesirable genes in a person’s cell by desirable ones brought in from the outside”) were “doubtful in the foreseeable future,” it claimed that human cloning was just over the horizon.^23^ The report also discussed at length the views of the “late geneticist H. J. Muller,” who was described as an “advocate of partial selection for the betterment of mankind.”^24^ Finally, it illustrated a variety of cloning scenarios depicted, among them the following:

In this way one could produce identical copies of the tens, hundreds or thousands of any person judged admirable: Mozarts, Shakespeares, Lincolns and Einsteins. Technically, it is still a long way from the use of frogs eggs to those of humans, but what can be done in frogs today will hardly not be possible in man tomorrow. […] Whether there would be general social acceptance for the practice of such radically new procedures in a foreseeable future is a question for which the sociologist and behavioral scientist will have to provide the answer. In any case it seems not too early to ponder the personal and social implications of success at the biological level.^25^

While parts of the NAS report were full of pathos, praising progress in the biosciences as both inevitable and socially desirable,^26^ there were also cautionary and critical voices. In the late 1960s, the question was raised as to whether political regulation of research in the life sciences was needed. Here, too, Lederberg played an important role. When his Stanford colleague, the biochemist Arthur Kornberg, announced the chemical synthesis of viral DNA in 1967, US newspapers widely reported on the breakthrough as “creating life in the laboratory.”^27^ Kornberg’s artificial synthesis of nucleic acid sequences and the first heart transplantation in medicine prompted the US Senate to consider more regulatory framework with regard to possible bioethical and social issues of modern life sciences. In February 1968, the Senator from Minnesota, Walter F. Mondale, initiated a joint resolution that called for the creation of a national commission with an ethical and political mandate (Waugaman 1969). This Mondale resolution was referred to the already existing Subcommittee on Government Research, which was asked to consider establishing a Presidential Commission on Health, Science and Society to discuss the “ethical, social, legal, economic and political questions” raised by these bioscientific developments.^28^ In particular, Senator Fred Harris of Oklahoma, chair of this subcommittee, stressed the responsibility of Congress to “identify and examine policy issues and to set guidelines.”^29^ Both Kornberg and Lederberg were invited to testify before the Senate’s committee in Washington, where both argued strictly against a “biological policy.”^30^ As the newspapers pointed out, the two Nobel laureates deployed all the weight of their scientific reputation to prevent political interference in research: “Scientist Doubts Genetic Abuse; Calls Research the Best Defense. Nobel Laureate tells Senate Panel a Federal Commission might hamper progress.”^31^

The extent to which genetic utopias also had an impact on public discourse in the United States can be seen, for example, in a 1969 *New York Times* article titled “The New Biology Taps Mysteries of Life.” The author, Robert Reinhold, described a range of situations, such as future preselection of a child’s sex, regeneration of damaged organs, cloning by “using preserved sperm and egg cells from superior individuals,” and “reprogramming cells” by genetic modification.^32^ His conclusion echoed the sense of inevitability long held by scientists like Muller and Lederberg: “The timetable differs somewhat according to which prophet you consult, but, for better or worse, the human race is moving inexorably into a new biological epoch.”^33^ But more dystopian views also circulated, such as in the article “The Frankenstein Myth Becomes a Reality: We Have the Awful Knowledge to Make Exact Copies of Human Beings” by bioethicist Willard Gailin,^34^ who contributed to the bioethical debate on cloning in 1972.

## The New Biology in West German Debates in the 1960s: Turning Utopia into Dystopia

The 1960s witnessed a range of sometimes conflicting intellectual movements. On the one hand, they were characterized by a modern faith in scientific rationality and technological progress. It was a period in which optimism (if not euphoria) about technology dominated many areas of science and society, especially cybernetics and research inspired by it, such as the emerging field of futurology. On the other hand, it was an era of intellectual and cultural movements that fundamentally questioned precisely these technological claims to dominance. After the atomic bomb and the horrors of National Socialism (both just two decades in the past), many philosophers fundamentally questioned techno-scientific or instrumental rationality and Enlightenment notions of progress. The concept of *technische Zivilisation* (technological civilization) and the question of the human being in the age of technology were central topics in German sociology and philosophy of the 1960s. While Muller and Lederberg’s utopian visions were driven by the unquestioned belief that technological and scientific progress would necessarily lead to social progress, their ideas were received in West Germany in the context of a fundamental questioning of scientific-technological claims to power. And they were, for the most part, rejected. Moreover, they and other so-called “Genforscher” (gene researchers) (Wagner 1970b, p. 8; 1970c, p. 33f.) were often labelled “Gen-Utopisten” (gene utopians),^35^ with a decidedly negative connotation (Portmann 1970 [1969]; Wagner 1964b, p. 1068).

It is true that, in comparison to the 1980s, there was no widespread *public* debate about the new biology in West Germany in the 1960s. However, general questions about the future of humanity in the age of science and technology were much discussed in intellectual and academic circles. The future of humanity was thus a central theme in Germany, too, a fact well exemplified by the *Darmstädter Gespräche*. Initiated in 1950, the Darmstadt conferences hosted leading philosophers, scientists, humanities scholars, sociologists, theologians, artists, and others at annual or biannual events to discuss challenges posed by the scientific and technological world. After symposia with topics like *Mensch und Technik* (Man and Technology, 1952), *Ist der Mensch messbar?* (Is Man Quantifiable?, 1958) and *Angst und Hoffnung in dieser Zeit* (Anxiety and Hope in this Time, 1963), the 1966 symposium was devoted to the theme: *Der Mensch und seine Zukunft* (Man and His Future) (Schlechta 1967). The second half of the 1960s in particular has often been regarded as a period of rapid cultural change. It witnessed the rise of a future-oriented optimism that began dominating several fields in society and politics and that also fueled the new scientific discipline of futurology. In 1966, for example, the weekly magazine *Der Spiegel* published an editorial on the subject “Futurology: The Future of Man Is Being Planned.”^36^ Elke Seefried has analyzed in great detail how the technophile visions of US futurologists were received in West Germany (Seefried 2015). Inspired by the public appeal of futurology and, above all, cybernetics (which was at its peak in Germany in the mid-1960s), a plethora of popular science books that presented visions of the future of man and society appeared in the mid-1960s. However, just as strong were critical voices that warned that the extreme acceleration of technological progress would lead to the downfall of civilization. Authors criticized cybernetics and the new biology as risky experiments, asserting that mankind was at stake. This is, for example, evident in the book *Experiment Menschheit* (Experiment Mankind), published 1967 by the Jesuit German popular science writer Paul Overhage. Over the course of 500 pages, he provided a critical analysis of possible consequences of the accelerated progress of scientific knowledge, which he argued would affect all aspects of human coexistence in the future:

The “human experiment,” the self-manipulation of mankind, has begun. It is already in full swing and has ushered in a radically new era in human history. In fact, research is striving to penetrate into the last structure of man and humanity, to reshape it and in this way (one is almost tempted to say) to virtually abolish man as we know him today, and to replace him with another kind of being. (Overhage 1967, p. 6)^37^

The “monstrous problem of humanity’s threat to itself” (Overhage 1967, p. 240),^38^ which since the 1950s had been seen above all in the danger of nuclear self-destruction, now also referred to fields such as medicine (overcoming aging and death), genetics (altering the genome), brain research (perfecting intelligence), and the threat to *Homo sapiens* posed by machine intelligence. Predictions about global crises such as overpopulation, world food crises and the nuclear threat fueled visions of a future with giant cities, a globally endangered ecological balance or overwhelming radioactive contamination (Overhage 1967).

In the context of both popular and academic discourses, holistic biologists and philosophers in West Germany were especially skeptical of developments in molecular biology. The triumph of molecular biology in the mid-1960s was a challenge to holistic conceptions of nature and an organismic biology that continued to be dominant in the biological sciences in West Germany in the 1950s and 1960s. As Christian Reiß (2023) has recently shown, holistic views of an organismic biology (which were rooted in the biological and epistemological debates of the interwar period) remained particularly influential in German popular science books. 39 In 1966, the year in which the international molecular biology community celebrated the deciphering of the genetic code, philosopher Helmuth Plessner (1892–1985) argued against what he called “Promethean culture,” in which purely scientific interest in knowledge was in danger of being corrupted by a “technical compulsion to produce” (“Fabrikationszwang”) (Plessner 2004 [1966], pp. 204–205). Plessner, who had been developing a holistic conception of biology related to philosophical anthropology since the 1920s, wrote in 1966:

With the conquest of outer space, and the purposeful unveiling of the secrets of the living substance and the mechanism of heredity, we have embarked on a path that would have filled the late 19th and early 20th centuries with horror if fantasies about it had seemed realizable to them for even a moment. For the supermen of science fiction have little in common with Nietzsche’s superhumans, who were conceived as an end, a conclusion and a fulfilment, as the crowning of the good, not as a spectre of a bad infinity. A Promethean culture has no limits and knows no fear. It is subject only to the law of boundless achievement and victory over all obstacles, a principle that has long since left its Faustian beginnings, if indeed it ever had them at all. (Plessner 2004 [1966], pp. 204–205)^40^

A statement by the elderly Martin Heidegger (1889–1976), who immediately associated the successes of molecular biology with the potential threat of “breeding of the human being,” pointed in a similar direction (Heidegger 2013 [1967], p. 124). The philosopher had previously developed an influential critical approach to modern technology and its implications for the very notion of humanity. In the early 1950s, he had analyzed the power of modern technology, understood as a technological world relationship, the momentum of which not only degrades nature to a resource but more fundamentally affects all ways of being and understanding the world. In a text from 1967, Heidegger declared that the technological *Eingeschlossenheit* (confinement) of humanity represented a cybernetic dominion (Herrschaftsbezirk) that would turn humans into nothing more than an element of feedback structures and deprive them of subjectivity (Heidegger 2020 [1967]). He saw molecular biology as a first step of a path leading to scientific-technological breeding of humans:

Biochemistry has discovered the scheme of life in the genes of the germ cell. This scheme, inscribed and stored as prescription inside the genes, is the programme of evolution. Science already knows the alphabet of this prescription. We speak of “an archive of genetic information.” On its knowledge is founded the firm expectation that one day *we shall be able to master the scientific-technological production and breeding of the human being*. The penetration of the genetic structure of the human germ cell by biochemistry and the splitting of the atom by nuclear physics belong on the same track, that of the victory of method over science (Heidegger 2013 [1967], p. 124, italics C.B.).^41^

In a German television interview two years later (1969), Heidegger explained that the new biology would be even more dangerous than nuclear physics, since the new science would start to construct and transform humans in line with social needs: “skilled ones and unskilled ones, clever ones and stupid ones,” (“Geschickte und Ungeschickte, Gescheite und Dumme”).^42^ This fear was formulated even more drastically by the physician and neurologist Karl Heinz Stauder (1905–1969), who had also penned novels and popular science books under the pseudonym Thomas Regau. In 1965, Stauder published his popular science book *Menschen nach Maß. Werkstoff Mensch im Griff einer seelenlosen Wissenschaft* (Customized Humans: Human Material in the Grip of a Soulless Science) in which he depicted a future aggressive world domination of modern genetics in dramatic ways:

We would gladly dismiss the geneticists’ views as utopian and uncritical if it were not for the fact that in this age of increasing speed, there are often only a few years between utopia and realization. So we have to face the aggressivity of modern genetics towards the world in order not to oversleep the hour of its realization. (Regau 1967, p. 29)^43^

Four main arguments arose out of this critical discourse in West Germany in the second half of the 1960s. First, molecular genetics research, which was already being referred to as “chromosome surgery” (“Chromosomen Chirurgie”) or “eugenic engineering” (“eugenische Ingenieurstechnik”), was often seen as a “twin” of modern nuclear physics (Regau 1967 [1965], p. 38, 48, see also Wagner 1964a). Authors pointed to the atomic bomb to illustrate the impending dangers posed by a biology that was undergoing a technological turn. Second, holistic biologists and philosophers criticized not only the reductionist view of man that they saw at work in modern science and technology, but also the reductionist methods of molecular biology itself. The “victory of method over science” also applied to the modern life sciences (Heidegger 2013 [1967], p. 124). Its adherence to technological rationality led some to compare the deciphering of the genetic code (as penetration of the cell nucleus) with nuclear fission. From their philosophical point of view, both were driven by a similar form of scientific-technological power without having sufficient knowledge of their consequences. Third, the 1960s debate grappled with the utopian—or dystopian—dimensions of biological science. Futurology was flourishing and prognoses were having their heyday. In this context, it was a widespread motif to refer to a “genetic utopia” (Wieser 1963, p. 321, 1966 , p. 13; Wagner 1964a, p. 334), and to argue that scientists’ obsessive visions of the future would surpass the wildest dreams of science fiction. Regau (i.e. Stauder), for example, argued that “it is one of the hallmarks of the era that the imagination of scientists has long since surpassed the imagination of novelists” (Regau (1967 [1965], p. 189; translation C.B.).^44^ In addition to this focus on the future, the debates also touched on the German past. Thus, fourth, the geneticists’ visions were seen as successors of Nazi eugenics.

## After the German Translation of the CIBA Symposium: Comparison of the New Biology to Nazi Eugenics

After the publication of the 1962 CIBA Foundation Symposium on *Man and His Future* was translated into German, Muller and Lederberg in particular came under fire from German critics. The conference had brought together twenty-seven scientists (including six Nobel laureates) to discuss “elements of a biological revolution” (Jungk and Mundt 1966, p. iii). The conference addressed issues such as the threat of overpopulation and resource shortages, advances in biology and technological revolutions, future developments in medicine, control of reproduction, genetics and eugenics, and the genetic modification of humans. The scientists shared the view that humanity was threatened by decline, which had to be countered by the new (and future) possibilities for intervention offered by the new life sciences. Above all, the elitist tone of the group’s discussions (which were published almost verbatim) left no doubt that the scientists saw themselves as the ones who should make decisions about (future) social issues, even if they went far beyond the realm of science. Critical responses to the CIBA symposium began to appear shortly after the symposium (Kaufmann 1964; Wagner 1964a; Wagner 1964b; Sigmund 1965; see also Petermann 2009). But the 1966 publication of the German translation of the talks accelerated the critical debate on genetic research in West Germany. The most unfavorable assessments compared the new biology with Nazi eugenics. Moreover, Muller and Lederberg were now often seen as the main proponents of the new biology, their ideas were taken representative of the dangerous developments it harbored. At the same time, Lederberg also became known to the German public when, in 1967, *Die Zeit* (an important German weekly) began publishing translations of some of the columns that Lederberg had written for the *Washington Post*.

Only a handful of geneticists contributed to the German debate. Among them were Friedrich Vogel, director of the institute of anthropology and human genetics at the University of Heidelberg, and Helmut Baitsch, director of the institute of anthropology and human genetics of the University of Freiburg. They took a critical stance on the potentially eugenicist applications of the new biology, addressed the serious ethical problems of genetic modifications of humans, and regarded ideas of a chromosome surgery and genetic modification of man as “purely utopian fantasies” (“blanke Utopie”) (Baitsch 1967a, p. 667; Baitsch1967b; Baitsch^1970^). Similarly, molecular biologist Peter Hans Hofschneider, director of the Max Planck Institute for Biochemistry, raised strong ethical concerns, and rather than dismissing human genetic modifications as fantasies, he claimed that they might one day become a problematic reality (Schlechta 1967, p. 75f; see also Salem 2013, p. 53). In 1969, a conference on genetics and society was held in Marburg. The *Marburger Forum Philippinum: Genetik und Gesellschaft* is often seen as the first conference in West Germany after 1945 that explicitly addressed issues of human genetics (Wendt 1970). On the one hand, this symposium might be seen as having been an attempt to give more objectivity to the discussion in Germany, especially after the CIBA symposium (Petermann 2009). On the other hand, the conference, which avoided discussion about Nazi eugenics, can also be seen as an attempt to cultivate public acceptance of genetic counseling and public awareness of the scientific potential for human betterment precisely by ignoring the past and brushing over continuities with Nazi eugenics. However, the publications of the sociologist Friedrich Wagner (1906-1974) probably had the greatest impact on the public debate in West Germany. In response to the CIBA symposium, Wagner described Lederberg and Muller as “prophets, even apostles of a genetic utopia that aims at controlling the human germ substance” (Wagner 1964a; Wagner 1964b, p. 1068). In 1969, he edited a widely discussed volume with the title *Menschenzüchtung* (Human Breeding). *Der Spiegel* soon published a detailed review of the book. The magazine emphasized that the eugenicist “extermination policy of National Socialism” had served as a warning to “German professors,” 45 making them reject the biological utopias of the geneticists in the United States. In fact, Wagner and the other authors directly compared the visions of Muller and Lederberg with National Socialist eugenics. This is all the more surprising given that German medicine and life sciences had, up to that point, hardly done anything to address their own culpability for the crimes of the Third Reich. It is a certain irony of history that Hermann Muller, the left-wing eugenicist who in the 1930s publicly took a stand against the so-called race hygiene of the National Socialists was now being placed in the tradition of Nazi eugenics (Paul 1984).

Wagner and most of the other authors—among them the Swiss biologist Adolf Portmann (one of the most prominent holistic biologists and natural philosophers at that time), Gerhard Helmut Schwabe (director of the Max Planck Institute for Freshwater Biology-Limnology), the theologian Karl Rahner and Wilhelm Küttemeyer, professor of anthropological medicine in Heidelberg—were all born around 1900. Thus, at the time of the book’s publication, all were in their mid-60s and mid-70s, and they had all experienced the National Socialist dictatorship as adults. Accordingly, the *Spiegel* framed the authors’ strong criticism of the “Gen-Utopisten” (“gene utopians”)^46^ as an appeal to an humanistic ethics. Wagner criticized the geneticists’ creation dreams and saw in them a new art of engineering that would degrade human beings to pure biomass. For him, the approaches of Muller and Lederberg were part of a long tradition of Social Darwinism. Muller’s “demiurgic fascination” with creating the “superhuman” (“Über-Mensch”) was, according to Wagner, rooted in his materialistic world view—and one can assume that Wagner also critically meant Marxist worldview (Wagner 1970c [1969], p. 16). Wagner urgently warned of a new biopolitical “state of gene control” (“genokratischer Staat”) and a new “era of euthanasia” that would devolve into “biological slums” (Wagner 1970c [1969], p. 37, pp. 45–46). The biologists Portmann and Schwabe were particularly critical of the underlying mechanistic, reductionist conception of nature and living beings that shaped the biological views of the US geneticists. For them, these concepts were clearly linked to a kind of anti-humanism (Portmann 1970 [1969], Schwabe 1970 [1969]). Küttemeyer painted the most drastic picture of an ongoing “biological revolution”, which the *Spiegel* review quoted in whole as representative of the book’s claims:

The mass experiment of National Socialism, carried out under the auspices of “science,” no matter how incomprehensible and horrific it may be, appears almost like a provincial event in comparison. What emerged in the heart of Europe back then has now taken hold in large parts of the world. What arose from a collaboration between state and research back then has now penetrated the very center of science. […] In the form of the most finely distributed terror in the sphere of influence of the modern superpower we call science, the “biological revolution” now threatens to become irresistible—with the help of a “scientifically” controlled global industry of consciousness (Bewußtseinsindustrie), which industrializes even the production of the human being (Küttemeyer 1970 [1969], p. 126-127; translation C.B.).^47^

## The Advent of Recombinant DNA Research: Overlapping Debates in the United States in the Early 1970s

When, in the early 1970s, the recombination of DNA in *E. coli* marked the beginning of the age of genetic engineering in the laboratory, the new public controversies arising around public health and occupational safety did not take place in a neutral space nor did they start from scratch. The 1960s utopian and dystopian discourses of a biological revolution had a lasting impact well into the 1970s, which is true not only for West Germany but also for the United States. In the early 1970s, the debate on the future of humanity in the biological age that had begun in the 1960s reached a high point, as did the early bioethical discussion of cloning and embryo research in response to it. At the same time, recombinant DNA technologies (using microorganisms and viruses as models and tools) emerged as a new scientific field. Although the researchers working on it, as well as their motivations, were completely different from the eugenicist project of human genetic enhancement, the general public perceived recombinant DNA research often as a kind of real-life manifestation of these utopias: “The once science-fictional idea of ‘genetic engineering’,” the *New York Times* reported on recombinant DNA research in 1975, “with its implications of altering the characteristics of human beings to ward off disease or to create armies of obedient slaves has taken a long step toward reality in the last two years. Biochemists have developed test-tube techniques allowing a much freer transfer of genes, the chemical units of heredity.”^48^ As a consequence, the older debates on the future of humans and the newly emerging debate on possible risks of recombination of DNA in microorganisms became intermingled. However, whereas the 1960s discourses on the biological revolution were dominated by broad moral issues and were mainly concerned with normative questions about intervening in the constitution of humanity, the discourses on biohazards that developed in the 1970s had a narrower focus. They involved technical questions about risks of laboratory experiments and the scientific and economic benefits of new biotechnologies, especially in the wake of the 1975 Asilomar conference.

It is important, however, to note that the 1975 *International Conference on Recombinant DNA Molecules* at Asilomar was *preceded* by an initial wave of ethical concerns as well as questions about the political regulation of the new life sciences in the United States. This is particularly clear in expert discussions in the field of science policy in the early 1970s. Only few examples are discussed in the following. In 1970, only two years after the aforementioned Senate hearing in the context of the Mondale resolution,^49^ the Advisory Council to the National Institute of General Medical Sciences (the NIGMS, a subdivision of the National Institutes of Health) organized a meeting on “Prospects for Designed Genetic Change.”^50^ The NIGMS had financed large programs in (human) genetics and what was called “genetic chemistry,” and the council (made up of twelve experts who regularly evaluated NIGMS research funding applications) thought it necessary to discuss developments in those fields that were “frequently referred to in the popular press as ‘genetic engineering’ as the final report stated.^51^

The experts discussed issues of human genetics and therapeutic methods, raised the possibility that a future with genetic manipulation might not be far off, and emphasized that the rapidly developing field of DNA research in molecular biology was in the “same stage that the atom bomb or atomic energy was in, say, 1920.”^52^ The state of the art of the molecular biology of viruses and bacteria as well as the first attempts of developing model systems for the delivery of a gene to a mammalian system were intensively discussed. The experts considered their future therapeutic and medical value and how they could be applied to human DNA.^53^ Although the Advisory Council’s members offered a mostly positive assessment of what they called “gene improvement” and “beneficial aspects of gene therapy,”^54^ they also addressed critical social and political issues. The biochemist Gordon Tomkins, professor at University of California, San Francisco, brought up the atomic bombing of Hiroshima. He was referring to political statements made to the *Washington Post* by molecular biologists Jonathan Beckwith and James Shapiro (whose research group at Harvard University had isolated the first (bacterial) gene in 1969). Although Tomkins distanced himself from the position of the Harvard colleagues, who had expressed deep concerns about the misuse of human genetic engineering by the state and had compared molecular biologists soon to be “regretful Oppenheimers” (Rasmussen 2014, p. 32), Tomkins warned: “So for the first time, large numbers of people are beginning to ask themselves: What are we doing?”^55^ Vasken Aposhian, professor at the department for cell biology and pharmacology at the University of Maryland, Baltimore, argued that the new loaded and offensive “term ‘genetic engineering’ has the revolting connotation to many, the scientific manipulation of the human race. […] But whatever happens with gene engineering is going to be the responsibility, I think, of scientists and politicians.”^56^ Comparisons between genetic engineering and the atomic bomb were not the only connections drawn at the meeting. Others repeatedly noted how human cloning might serve as a striking example of how the social responsibility of scientists was addressed in society. “It may only be a matter of developing technology to devise methods to clone a human being from a selected human cell,” the geneticist and pioneer of pharmacogenetics, Arno Motulsky from the University of Washington in Seattle, emphasized, and he moved on to the possibility of a research moratorium:

Thus it might be possible for a dictator to create groups of soldiers. Or, a society might want to create highly intelligent groups for certain tasks such as devising better weapons. The question naturally arises: Should a moratorium on this kind of research be declared? I think this would be very difficult. My feeling is these techniques will be used medically and abuses in certain instances will be difficult to prevent.^57^

Indeed, in the early 1970s, the public debate about cloning human beings and experimental research on embryos had reached an apex. In 1971, the 12th meeting of the Panel on Science and Technology of the Committee on Science and Astronautics of the U.S. House of Representatives focused on “Potential Consequences of Experimentation with Human Eggs.”^58^ In his testimony for the panel, the molecular biologist and Nobel laureate James Watson warned of the imminent possibility of the cloned human. He argued for science regulations, pleading that “this is a matter far too important to be left solely in the hands of the scientific and medical communities. The belief that surrogate mothers and clonal babies are inevitable because science always moves forward, an attitude expressed to me recently by a scientific colleague, represents a form of laissez-faire nonsense dismally reminiscent of the creed that American business if left to itself will solve everybody´s problems.”^59^ Even the *Wall Street Journal* picked up issues of human cloning and gene therapy. Reporting about research at the National Institutes of Health, the journal ran the headline in 1971: “Some Scientists Seek to Alter Human Gene to Cure Many Diseases—but Critics Claim Research Could Lead to the Breeding of Physical, Mental Giants.”^60^

Questions about the regulation of science were posed especially by scholars from a range of disciplines working in bioethics, which was becoming increasingly institutionalized in the United States at that time. In 1972, the National Presbyterian Center, the Board of Christian Social Concerns of the United Methodist Church and the Episcopal Cathedral of the Diocese of Washington funded the publication of a widely received multi-author volume on *The New Genetics and the Future of Man*. The book’s contributors included scientists like Leon Kass, philosophers like Daniel Callahan and Joseph Fletcher, lawyers like Alexander Capron and theologians. These thinkers discussed in detail the moral and ethical issues posed by the new biology, particularly with respect to the beginnings of life and gene therapy (Hamilton 1972). In an article titled “The New Biology: What Price Relieving Man’s Estate?,” published in *Science* in 1971, Kass, then executive secretary of the Committee on Life Sciences and Public Policy of the National Academy of Sciences, called for political control of research. He interpreted the new biomedical technologies—among them cloning, “laboratory fertilization of human egg,” and genetic engineering—as the “advent of ‘human engineering’” that bore the risk of “dehumanization” (Kass 1971, p. 779f., p. 783). Kass attacked those who argued against political regulation of science as ignorant “proponents of laissez-faire” (Kass 1971, p. 787). For him, “the question [was] not control versus no control, but rather what kind of control, when, by whom, and for what purpose” (Kass 1971, p. 787). He addressed not only policy issues in the United States, but also the global dimension of the problem:

Means for achieving international regulation and control need to be devised. Biomedical technology can be no nation’s monopoly. The need for international agreements and supervision can readily be understood if we consider the likely American response to the successful asexual reproduction of 10,000 Mao Tse-tungs. (Kass 1971, p. 787)

As these quotations show, by the 1970s there were already calls for greater control of biotechnological research in the air. However, many influential scientists rejected any demands to regulate scientific research. For example, in 1971 Joshua Lederberg posed the rhetorical question: “Should we spend much time worrying about the ethical implication of the genetic findings of the next century, when we must do this on the basis of a set of assumptions about the human condition that will surely change dramatically in every other way?”^61^ In the same year, the physicist and editor of the journal *Science,* Philip Abelson*,* argued that the “anxiety about genetic engineering” was completely unjustified:

During the last several years, the public has repeatedly been warned that science is creating additional problems through raising the possibility of test tube babies and ‘genetic engineering.’ The response of the public has been negative, with some calling for a halt to research in molecular biology […] Talk of the dire social implications of laboratory-related genetic engineering is premature and unrealistic. It disturbs the public unnecessarily and could lead to harmful restrictions on all scientific research. (Abelson 1971, p. 285)

Others, like Salvador Luria—an Italian-born molecular biologist who was forced to emigrate from fascist Europe in the 1930s and who received the Nobel Prize for his pioneering research on bacteriophage in 1969—were more cautious and emphasized the limitations of scientific research. In a critique of what he perceived as an “antiscientific attitude” that harbored distrust and even “hostility of the general public towards science” (Luria 1972, p. 355), Luria argued against the widespread view of a value-free science and he asserted that scientists have special responsibilities. Arguing that not every form of research is legitimate, especially if it affects human beings, he saw society as the authority that should decide which technologies it wanted to use in the future.

The Australian bacteriologist and immunologist Sir Frank Macfarlane Burnet, another Nobel laureate, was even more cautious. Long before molecular biologists began calling for a moratorium on specific experiments in the newly emerging field of recombinant DNA research in 1974, McFarlane spoke out at the 1971 Pacific Science Congress (an international conference with more than 1,000 participants) for a stop to certain areas of genetic research on viruses and bacteria. In October of that year, the *Medical Tribune* reported:

Peril seen in Studies on Microbe Genetics: Nobel prize winning virologist and immunologist Sir Macfarlane Burnet called here for a world-wide halt to all experimental research into the genetics of potentially pathogenic bacteria and viruses. He warned […] that the world is in danger of accidental genocide arising from this type of research. Sir Macfarlane, who was giving the presidential address, at the congress, said: “There is only one source from which devastating new infectious diseases could come. If they come, they will come from deliberate or accidental manipulations in laboratories dealing with microbial genetics.”^62^

Other reports, however, attempted to counter critical assessments with the studies’ potential medical benefits. From the media reports it becomes clear how widespread the notion of *genetic engineering* was already in the early 1970s—a term often considered problematic by scientists because of its negative connotation which would “tend to de-emphasize the humanistic potential of this field for contributing to human health.”^63^ Terms such as *human engineering* (Kass 1971, p. 779ff.) or *human genetic engineering*, also widely used, had even more dystopian connotations: On the occasion of the twentieth anniversary of the discovery of the DNA double helix, the journal *Medical World News* felt compelled to emphasize the medical benefits of the new biology against the widespread dystopian view of it as a form of “human genetic engineering,” a term that had been “coined in response to the DNA revolution.”^64^ In May 1973, the journal published an extensive article on the current state of research in both human genetics and molecular biology that proclaimed: “Human Genetic Engineering—No Brave New World but Brand New Medical Potentials.” In contrast to “some disturbing suggestions about what ‘human genetic engineering’ might soon achieve,” an issue that would be “widely discussed by layman and scientists alike,” the journal offered a positive view of the diverse therapeutic and medical applications of DNA research.^65^

This was the situation at the beginning of the 1970s, before Asilomar. There was already intense debate about genetic research on humans, and questions about research control had already been raised with regard to cloning and embryo research. The years 1972 and 1973, however, have often been depicted as representing a turning point in the history of biology, when the first results of a recombination of DNA in *E. coli* were published and widely reported on. A research group at Stanford University led by biochemist Paul Berg had developed molecular techniques to join together DNA from different sources. They generated hybrid DNA by integrating DNA from *E. coli* and the phage lambda into the DNA of the SV40 (a primate tumor virus) with the aim of inserting this hybrid DNA into eukaryotic cell cultures (Jackson et al. 1972). In 1973, another group of Californian scientists—Stanley Cohen from Stanford University and Herbert Boyer from the University of California—were able to show that the recombination of genes could be successfully integrated into a bacterial genome (using plasmids as vectors) and that these recombinant DNA molecules were propagated by the bacterial host (Cohen et al. 1973). This marked the birth of what came to be known as gene cloning (Yi 2015, pp. 83–112). As has been recounted many times, these findings led to intensified discussion on the possible risks of the new field of genetic engineering within the molecular biology research community. As a result of the Gordon Conference on Nucleic Acids in June 1973, the molecular biologists Maxine Singer (NIH) and Dieter Söll (Yale University) published a letter that was sent to the president of the National Academy of Sciences, Philip Handler, in order to establish a committee on risk issues (Singer and Söll 1973). A year later, in July 1974, the members of this committee (eleven molecular biologists, chaired by Paul Berg)^66^ drafted a letter calling for a worldwide moratorium on certain genetic engineering experiments (Berg et al. 1974). This quickly led to a sweeping media debate about genetic engineering, and the Berg letter was often portrayed as a unique historical instance of scientists exerting voluntary self-restraint. At the same time, Paul Berg, Maxine Singer and a few others were organizing the *International Conference on Recombinant Molecules* in Asilomar, to be held in February 1975.^67^ In the post-Asilomar statement, the research moratorium was lifted (Berg et al. 1975). The scientists gathered at the conference put forward a sketch of potential guidelines for research on genetic engineering and laboratory safety, which was further developed by an NIH commission in the following months. The subsequent events have also been widely studied. Susan Wright has shown that the first phase of the discussion about risk assessment, initiated by the scientists themselves, was quickly followed by a second phase after 1975 in which the guidelines were progressively weakened. Wright describes this as a shift from a discourse of “prevention” to one of “dismantling controls” (Wright 1994, pp. 337–382). This second phase also includes what is known in the United States as the recombinant DNA controversy, which peaked in 1976-1977 with the well-known conflict over issues of laboratory safety that pitted Cambridge City Council and Cambridge citizens against Harvard and MIT scientists (Goodell 1979; Krimsky 1984).

In the course of these post-Asilomar debates, which were widely reported on in the US media in the period from about 1975–1977, the focus shifted significantly. The debate no longer centered on the moral or bioethical question of the scope and limits of possible future genetic engineering interventions in human nature, which had been central at the beginning of the decade, but rather on the legal and political issues of regulating biotechnological research as well as controversial questions about public involvement in these decisions and the autonomy of scientific research. The dangers of a possible future “human genetic engineering”^68^ faded in significance as concerns grew about the real and perceived risks of an unintended release of genetically modified, pathogenic microorganisms and the resulting threat to the population, for example in the form of an epidemic. However, the lasting effect of the former debates on human engineering was also evident, especially in the public sphere. In a letter that a concerned citizen, Kenneth Quade from Pembine, Wisconsin, wrote to Paul Berg in February 1975, shortly before the Asilomar conference, it was emphasized that scientists like Watson, Kass, Callahan and others had warned in the early 1970s “that the public should begin to discuss the moral, ethical, legal and political implications of genetic engineering.” Quade wanted to get answers from Berg with respect to the following:

I am asking you and your conference on genetic manipulation to be held at Pacific Grove, California on February 24–27 to either repudiate or substantiate publicly or at least by a personal letter to me the following statements: 1) It is possible human life could be produced completely in the laboratory by either a combination of the test tube and artificial womb or by cloning by 1980. 2) It is possible some country or countries will be producing more human life from the laboratory than from the womb by the end of this century.^69^

Berg’s handwritten response to the letter gets to the point of how the Asilomar conference also served to give public debates a more concrete and, compared to the early 1970s, narrower focus: “Sorry, but these matters are not the subject of the conference later this month.”^70^

Dystopian ideas about cloning and genetic enhancement of humans, however, sometimes still fueled public protests. In April 1977, *Time* magazine reported on a campaign launched by those opposed to genetic engineering. At a forum of the National Academy of Sciences in Washington, “unruly opponents of genetic research, chanting ‘We shall not be cloned’ took over the stage and unfurled a banner reading ‘We will create the perfect race – Adolf Hitler’.”^71^ The National Academy of Sciences had organized a three-day forum in order to discuss issues of research regulation in March 1977. The opening session was disrupted by a group of citizens and lay activists, led by Jeremy Rifkin. At the same meeting, the formation of the *Coalition of Responsible Genetic Research* was announced, including eminent scientists and environmentalists (among them the Nobel prize winners George Wald and Sir Macfarlane Burnet), who worried about the impact of commercial interests on research and who still argued for a world-wide ban on genetic engineering (Yi 2025, p. 41; Wright 1994, pp. 224–225).

## From the 1960s to the Early 1980s: Discursive Shifts and Debates on Recombinant DNA Research in West Germany

As discussed above, the 1960s debates about the new "biology and the future of man" had a lasting impact on how developments in DNA research were perceived by the public in the 1970s. However, the 1970s also witnessed significant changes in how these discourses were framed. Three aspects should be emphasized.

First, a new, younger generation of molecular biologists were primarily responsible for the advancement of recombinant DNA research in the 1970s. Often influenced by the political movement associated with 1968, they no longer wanted to have anything to do with eugenics. The same applies to a new generation of critics. In Germany in the 1960s, the harshest critics of the new molecular biology were generally holistically oriented, often conservative philosophers and biologists in West Germany who had themselves experienced National Socialism and who were anything but leftists. However, in the course of the 1970s, a new generation took over. During the 1970s and early 1980s, most criticisms of genetic engineering developed out of the new social movements, which were to the political left. In particular, the anti-nuclear movement had a significant influence on public criticism of genetic engineering in the early 1980s (Radkau 1988). But the environmental and peace movements (the latter reached a peak in West Germany in the early 1980s) played an important role here, too. And last but not least, criticism of genetic engineering and reproductive medicine was particularly strong in the West German feminist movement in the late 1970s and early 1980s.

Second, while the 1960s still represented the postwar economic boom and was characterized by optimistic views of social and economic progress, the 1970s saw a sharp cultural turn away from optimism. Several historians have described the decade as a crisis-ridden historical turning point that finally put an end to the postwar boom period (Bösch 2019, Doering-Manteuffel and Raphael 2012, Ferguson et al. 2011).^72^ Thus, amidst the diversity of political and economical discourses in the 1970s, a common theme was the loss of utopia as a referential paradigm. This loss of utopia was also evident in scientific discourses, especially in the growing public debates on genetic engineering. These debates were in part driven by a general sense of crisis in popular media. But perhaps more importantly, the arrival of genetic engineering was no longer seen as something distant in the future, but as a real situation that was confronting humanity in the here and now.

Third, the political valence of the imaginary changed fundamentally over this time period. In the 1960s, critics of the new biology had warned that the gene utopians’ visions of the future were akin to science fiction. But in the 1970s, the argument was turned on its head: Now it was the molecular biologists who accused critics of genetic engineering of concocting science fiction nightmares in their warnings of the possible risks of recombinant DNA research. In an expert hearing before the Attorney General of the State New York in 1976, James Watson, for example, provocatively raised the question: “Recombinant DNA research—are we proposing regulations over imaginary dangers?”^73^

In fact, media reports from the 1970s, especially in West Germany, reveal a subtle affinity with narratives of artificial humans that originated in science fiction stories, and these reports reflected this mood of crisis. Although public debate on these issues was not yet very significant in West Germany in the 1970s (Brodde 1992), German media did report sporadically on the new developments in genetics, molecular biology, IVF research, and cloning research. A closer look at the reporting of outlets like *Der Spiegel* shows how strongly this debate was shaped by a fundamental sense of crisis. In 1970, *Der Spiegel* published a feature about a number of new developments in the biosciences. The magazine’s cover showed a naked female body in a test tube with the title: “Biochemistry: The Human Being is Being Rebuilt”.^74^ References to “Frankenstein experiments” abounded,^75^ as did long, detailed discussions of Friedrich Nietzsche’s concept of the *Übermensch*. As *Der Spiegel* stated in 1970, “the creation of the superhuman became the goal of sober science.”^76^ Huxley’s *Brave New World* was a standard reference in articles on a variety of developments in the life sciences during the 1970s. These reports often lumped together research in the field of IVF, cloning research on frogs and developments in genetics, and genetic engineering, as evidenced by the titles of *Spiegel* articles. From the late 1960s to the early 1980s, the magazine carried headlines like “Hundreds of Heroes” (1968), “Straight to Hell” (1970), “A City Full of Marilyn Monroes“ (1973), “Genetics: A Thousand Times Worse than Hitler” (1978), “A Step Towards Homunculus” (1978), and “Genetics: Dodecuplets, Lots of Dodecuplets” (1981).^77^ Several articles featured illustrations of the homunculus myth in alchemy and modern mutants from pop culture, thus following traditional cultural-historical patterns, reproducing ideas about the artificial creation of human beings, and dramatically bemoaning the potential of the new life sciences to de-humanize mankind. These reports give an idea of the context in which the new results of recombinant DNA research were received by the public in the 1970s.

However, *political* debates on the regulation of genetic engineering in Germany did not begin much later than they did in the United States in the 1970s. After the 1975 Asilomar Conference on recombinant molecules, Germany, too, saw the formation of an expert discussion about the potential risks of recombinant DNA research. Questions about the safety standards for laboratories and the political necessity of regulating this research were also raised in West Germany. Similar to the NIH guidelines in the United States (and comparable regulations in the United Kingdom), *Richtlinien zum Schutz vor Gefahren der in vitro Neukombination von Nukleinsäuren* (guidelines for protection against the hazards of in vitro recombinant nucleic acids) were negotiated and enacted in Germany between 1975 and 1978. Moreover, the *Zentrale Kommission für Biologische Sicherheit* (Central Commission for Biological Safety) was established as an expert body in 1978 (Klassen in press; Gottweis 1998). This scientific-political expert discussion came to a head after 1978, when the Federal Minister for Research and Technology sought to establish a federal law regulating research in genetic engineering (Gentechnikgesetz)—an approach that failed due to fierce resistance from research organizations such as the Max Planck Society (MPG) and the German Research Foundation (DFG) in 1980 (Klassen in press; Schwerin et al. 2023).^78^

This expert discourse was quite similar to its US counterpart, although there was one fundamental difference: from 1974 to 1978, the wider public was not yet involved to the same extent as in the United States. Over a longer period, these debates were more or less held behind closed doors at the Federal Ministry of Research and Technology (Bundesministerium für Forschung und Technologie, BMFT) and scientific organizations. Germany lacked the kind of broader critical public interest in recombinant DNA that found expression in initiatives like the Cambridge Experimentation Review Board in the midst of the U.S. controversies.^79^ Still, in an expert panel initiated by the science journal *Bild der Wissenschaft* in 1977, Federal Minister for Research and Technology Hans Matthöfer regretted that Germany lacked a movement comparable to Science for the People and called for scientists to get more engaged on issues of public concern.^80^ However, all that changed significantly in the years after 1978 with the announcement of the birth of the first child conceived by in vitro fertilization in Great Britain.

The field of reproductive medicine attracted extraordinary media attention. The birth of the first so-called test tube baby (in German *Retortenbaby*, which has strong negative semantic associations with the idea of a *homunculus* in alchemy) was widely perceived in the media as a watershed moment. It was also a turning point for the debate on recombinant DNA research. The fertilization of human egg cells outside the body—and the concomitant possibility of manipulating human embryos—opened up a future horizon of possible applications for genetic manipulation in humans. For the critics, the dystopia of genetically modified humans, which had been discussed since the 1960s in terms of eugenicist optimization, barged into the real world. The development of IVF thus not only represented a significant turning point in the field of assisted reproduction, but also lent new urgency to the question of the social impacts and risks of recombinant DNA techniques. In turn, this raised fundamental ethical and anthropological questions about embryo research that went far beyond the debates among scientists about risks of the early 1970s, which had been limited only to aspects of laboratory safety.

The German Federal Ministry of Research and Technology and politicians formulated their own responses to the changed situation. Beginning in 1978 and reaching a highpoint in the early 1980s, a number of different committees were convened that focused on how to regulate research in these new fields of life sciences, which meant both recombinant DNA research as well as embryo research. For example, in October 1978, the Committee for Research and Technology of the Bundestag convened an expert hearing on gene research, and in September 1979, the German Federal Ministry of Research and Technology organized a conference with international molecular biologists on *Chancen und Gefahren der Genforschung* (opportunities and risks of gene research) (Herwig and Hübner 1980). The “ethical and legal problems of the application of cell biological and genetic engineering methods to humans” was the subject of another expert hearing that took place in 1983 (Bundesminister für Forschung und Technologie 1984). Finally, in 1984, the Bundestag initiated the *Enquete Kommission Chancen und Risiken der Gentechnologie* (Enquete Commission on Opportunities and Risks of Gen Technologies)*.* Consisting of experts from various fields and politicians from all parties represented in the Bundestag, the committee worked intensively for over two years to compile a comprehensive inventory of the social and economic opportunities and risks of genetic engineering. The aim of all these committees was to advise parliament on the question of the need for laws regulating genetic engineering and research on human embryos (Catenhusen and Neumeister 1987; see Klassen in press, Jasanoff 2005; Gottweis 1998).

At the same time, the public debate underwent a dramatic shift. In the early 1980s, genetic engineering became a controversial topic, which was now being discussed by a wide range of actors from civil society, such as churches, trade unions, industry and science associations, and, most of all, the new social movements (Radkau 1988). As a greater range of actors began engaging more deeply with these issues in the early 1980s, criticisms of genetic engineering became more nuanced (Klassen in press). But despite the rise of a more complex debate about the social and ecological risks as well as looming commercialization of research of the new life sciences, the old narrative of creating artificial life and images of artificially created man remained central themes in the public debate in West Germany. A few examples from very different contexts might serve to illustrate this: In 1978 Jost Herbig, a popular science writer, published *Gen-Ingenieure: Durch Revolutionierung der Natur zum neuen Menschen?* (Genetic Engineers: Through a Revolution of Nature to the New Man?) (1978). The book was primarily a critique of the dynamics of a newly emerging bio-economy. It also dealt in detail with the risks of accidentally releasing genetically modified microorganisms and focused on other concrete ecological risks. However, it also engaged with the hubris of the scientist becoming a creator and deeply rooted cultural and historical images of artificial human creation. “The age of synthetic biology has begun. Engineering and biology unite in genetic engineering [...] Biologists become creators” (Herbig 1978, p. 9). A conference of women against genetic engineering and reproductive medicine, held in April 1985, took a similar position. The first page of the published documentation of the conference features visual references to alchemy and the *homunculus* narrations, and on the next page, a long quotation from Goethe’s *Faust* describes the scene where Wagner creates a homunculus (Die Grünen et al. 1986).

The Austria-born biochemist and pioneer of DNA research, Erwin Chargaff, also drew a direct dystopian connection between genetic engineering and reproductive medicine. Chargaff was forced to migrate when the Nazis came to power in 1933 and had worked as a biochemist throughout his life in the United States. He became one of the leading critics of genetic engineering in the scientific community. In 1976 he published a widely read critical letter in the journal *Science*, in which he accused his colleagues of neglecting the ecological and evolutionary risks of recombinant DNA research (Chargaff 1976). But while he was considered more of an outsider figure by his colleagues in the United States, Chargaff rose to become one of the most authoritative figures on the subject in the German debates. When recombinant DNA research came into being, Chargaff asserted (like Heidegger did in the 1960s) that both physics and biology had corrupted the ethos of scientific research with their nuclear fissions (atomic nucleus and cell nucleus) and warned of the consequences. His views illustrate how much the cloning debate, which scientists had been discussing since the 1960s, continued to resonate well into the 1980s. In an interview from 1984, Chargaff saw the danger of “genetic manipulation” in the “brutalization of the human imagination.”

After a while, anything becomes possible. What is feasible is not only done, it *has to* be done. It becomes an imperative. [...] I hold genetic engineering responsible for the excesses of reproductive research, which is now producing these abominations like test-tube babies, frozen embryos that may even be available for purchase at a store. It is the complete industrialization of processes that have always been sacred to humans. Once the ethos or moral concerns of the people (Volk), of the world, have been eroded, then from a certain point on anything becomes possible. [...] The excesses of this thinking then lead to the idea of “cloning”: the “clone” as a complete copy of the father. In mice, it has supposedly already been achieved in theory. I have my doubts about it.^81^

## Reactions from Scientists: Imaginary Risks and Emotional Debates

Against the charged discourses of the late 1970s and early 1980s, leading German molecular biologists tried to objectify the debate. The era witnessed a stark increase in political expert hearings, and a handful of prominent molecular biologists, especially Peter Starlinger (professor of molecular genetics at the University of Cologne) and Peter Hans Hofschneider (director at the Max Planck Institute for Biochemistry in Munich and one of the co-founders of the first US-European biotech company, Biogen, in 1978) became well-known scientific experts in the political arena. Indeed, most German molecular biologists working in the field of recombinant DNA tried to publicly refute the widespread view of genetic engineering as a fundamental turning point in the history of biology or as the advent of a new, apocalyptic epoch or “genetic age” (Flöhl 1985, p. 375). For them, their research did not represent a qualitative leap of biology into technology. Instead, they saw recombinant DNA research on *E. coli* as part of a long tradition of biotechnological uses of microbes. Many scientists sought to naturalize their (new) technical approach of recombining DNA in plants or microbes by claiming that the recombination of genes in the laboratory represented a kind of acceleration of processes that would take place in nature anyway. Finally, they did not see their own molecular genetic modifications of microbes or plants as a potential risk. In public statements, they argued again and again that people should separate their genetic engineering research on microbes from work that might directly affect human beings, such as embryo research, cloning, and reproduction research. Most molecular biologists rejected the dystopian comparisons of the new bioscientific field with the potentially apocalyptic consequences of modern physics common among critics during the heyday of the anti-nuclear movement in Germany. For example, in 1979, Starlinger, himself an activist in the peace movement against nuclear weapons, stated at the conference on *Chancen und Gefahren der Genforschung* (Opportunities and Risks of Gene Research) that was organized by the German Federal Ministry of Research and Technology:

I think we have to distinguish between fundamentally different things in the discussion of risks. One is the discussion of risks that can be clearly postulated and, if necessary, falsified. And the other is the science fiction–like risk discussion.^82^ (Herwig and Hübner 1980, p. 124; translation C.B.)

Scientists argued that the blurring of boundaries between fact and fiction prominent in the media and science fiction of the time (Brandt 2007, 2009b) contributed to the fact that purely speculative ideas were discussed in earnest even though they had nothing to do with real science. They regularly complained that the public’s reaction to genetic engineering was overly emotional and irrational. Scientists often attributed the lack of acceptance of the new genetic engineering among the public to a lack of (technical) scientific education. Thus, the concept of risk that shaped public debate diverged sharply from that shared by most molecular biologists. Molecular biologists distinguished what they saw as purely speculative, hypothetical risks, sometimes referred to as science-fiction, from real risks such as those posed by nuclear energy. These different concepts of risk and different epistemic views of science highlighted the incommensurability of the deeper epistemic understanding of what was actually being discussed on each side. For the scientists, the emotional character of the public debates demonstrated a lack of scientific rationality. For the critics, however, the scientists’ unwillingness to question their own basic assumptions was a sign that there was an urgent need for philosophical or sociological analyses and reflections on the very notion of scientific rationality itself. From these disagreements about what constitutes scientific rationality emerged a fundamental cultural change in the way society deals with science and technology, a change that the sociologist Ulrich Beck described in the 1980s as “disenchantment” with the truth and validity claims of science and a “demonopolization of scientific claims to knowledge” (Beck 1990 [1986], p. 254, 256). In 1986 he wrote: “The fear of the ‘progress’ of genetic engineering is widespread today. Hearings are taking place. The churches protest. Even scientists who believe in progress cannot shake off the creeps” (Beck 1990 [1986], p. 329, translation C.B.). However, according to Beck, these emotional debates were signs that Western societies were entering a *reflexive modernity*—a new phase in modernity that questions traditional views about the autonomy of science and places the demarcation between science, politics and society up for renegotiation.

## Conclusion

This article has analyzed continuities and shifts in the public debates about genetic engineering in the United States and West Germany from the 1960s to the early 1980s. This time period witnessed the development of new, more nuanced conceptions of risks. But on a more fundamental level, the debate about recombinant DNA technologies was just one part of a much broader discourse on reflexive modernization that was inextricably linked to a profound debate about values (Beck 1990 [1986]). Heated debates about the future of humanity in a world driven by science and technology arose as early as the 1960s in both the United States and West Germany. Scientific developments such as molecular biology, cloning research, and research into embryos and reproduction were often considered to constitute a new biology revolution. To understand these debates, it is necessary to consider them in the broader historical contexts of shifts and continuities of early 20th-century eugenics, as well as other contemporary scientific and social developments of the 1960s, such as futurology and concerns about the future of humanity in the technological age. These debates essentially revolved around different values and different views regarding the socially legitimate scope of, or moral limits to, future biotechnological changes to humans. At the core of these debates were utopian or dystopian views of “Menschenzüchtung” (“human breeding”) (Wagner 1970a) or a “human engineering” (Kass 1971, p. 779f.)—terms that had been in circulation prior to the breakthrough in, and widespread discussion of, recombinant DNA research. However, these debates took place in very different circumstances in the United States and West Germany. In the 1960s, leading US scientists and scientific organizations discussed “human genetic betterment” (Muller 1965, p. 100)—and even the cloning of human beings—with a positive outlook. The vision of the *new man*, the optimized human being of the future originally developed by early 20th-century eugenics, was discussed in a new way, especially by scientists. Their visions of the future were shaped by the unbroken narrative of scientific progress and technological euphoria that were typical of the intellectual climate in certain fields in the 1960s. In contrast, debates in West Germany took a different direction. In light of the past Nazi eugenics, these visions of the future were strictly rejected as science fiction-like dystopias.

Science policy issues were also raised much earlier than in the context of the 1975 Asilomar conference. In response to developments in the life sciences in the late 1960s, politics began to address the question of political responsibility for regulating research in these new fields in the United States. The scientific utopias of the 1960s had an afterlife in the 1970s and 1980s. Their interpretation as dystopian nightmares had a lasting impact on fears about the danger of creating genetically manipulated humans, which many assumed was a goal that scientific research would pursue. The article has explored these discursive complexities and dynamics, it has traced how debates on human cloning and embryo research, as well as the emerging bioethical debate in the United States, intersected with the newly emerging rDNA debate in the first half of the 1970s. While the Asilomar conference undoubtedly opened up a new discourse in the mid-1970s, it also narrowed former ones. The rapidly developing recombinant DNA technologies of this period finally shifted the focus of public controversy. Fears of biohazards (primarily laboratory based hazards, caused by an unintentional release of genetically modified microbes or pathogens) and the differing perceptions of risks held by scientists and the public were now at the heart of the debates, which had shifted from utopian or dystopian ideas about the future of humanity in a genetically engineered world to the concrete risks posed by emerging genetic engineering technologies. In the United States in particular, the rather morally charged debates of the 1960s and early 1970s evolved into a political controversy about biohazards and scientific autonomy in the 1970s.

After the Asilomar conference in 1975, a political discussion about regulating the new life sciences began also in West Germany. Scientific and political experts discussed the need for laboratory safety guidelines for recombinant DNA research, which were also being developed in the United States around the same time. In the mid-1970s, these political negotiations were still being held out of public view in expert hearings and discussions between politicians, scientists, research organizations, and the German Federal Ministry of Research and Technology (BMFT). Beginning in the late 1970s, however, the public debate intensified, reaching a peak in the mid-1980s. In West Germany, the political controversy about recombinant DNA technologies remained inextricably intertwined with debates on cloning, embryo research and IVF up into the early 1980s. Moreover, other discourses such as philosophical critiques of technological rationality, political critique of emerging (bio)capitalism, the political ecology movement, and, not least, arguments from theology and moral philosophy, developed heterogenous critical perspectives. However, the existential threat to humanity posed by the new life sciences remained a key topic of public debates in West Germany, unlike the United States.

## Data Availability

No datasets were generated or analysed during the current study.

## Footnotes

1 Original: “Ob die Genforschung Fluch oder Segen mit sich bringt, hängt aber nicht nur von der Geschwindigkeit der Entwicklung, sondern in viel stärkerem Maße von den Zielen ab. Diese sind, da wir erst am Anfang des Genzeitalters stehen, allenfalls in den Umrissen zu erkennen. Deshalb läßt sich die Frage: ‚Genforschung– ‚Fluch oder Segen?‘ gegenwärtig nicht so entschieden beantworten, wie sie gestellt worden ist. Nach der Lektüre dieses Bandes kann aber kaum Zweifel daran bestehen, daß der Mensch in seinem Wesen noch nie so bedroht war, wie durch die Gentechnik.”

2 The following scientists from West Germany participated at the Asilomar conference: Walter Goebel, *Gesellschaft für Molekularbiologische Forschung*, Braunschweig, Gerd Hobom, University of Freiburg, Peter Hans Hofschneider, Max Planck Institute for Biochemistry, Munich, Peter Starlinger, University of Cologne, Hans Zachau, University of Munich, John Tooze, European Molecular Biology Organization, Heidelberg. Sixteen journalists participated, all of them from U.S. newspapers or international science journals. Rainer Flöhl of the *Frankfurter Allgemeine Zeitung* was the only journalist from a non-English language newspaper; see *Report of the Organizing Committee of the Asilomar Conference on Recombinant DNA Molecules*, Paul Berg papers, Stanford University Archive, SC0358 ACCN 1989-062, Stanford, California. (hereafter abbreviated to, Berg Papers), Box 13, Folder “Photocopy of the original final report.”

3 On the history of the Asilomar conference and the emerging rDNA controversy from the perspective of historical actors see (e.g. Watson and Tooze 1981; Krimsky 1984; Fredrickson 1991; Berg and Singer 1995. For recent historical analyzes see Campos and Cassata 2025; Campos 2025; Cobb 2025, 2022 (Chaps. 2– 4); Yi 2015, 2025; Scheffler 2025; Cassata and de Chadarevian 2025; de Chadarevian 2005).

4 “Human genetic engineering. No brave new world but brand new medical potentials,” *Medical World News*, May 11, 1973, p. 45–57, quotation p. 45; newspaper clipping, without author, Berg Papers, Box 14, Folder: “News articles prior to 1974.”

5 “Code Breakers Matthaei and Nirenberg: Cracking the Genetic Code,” cover of the *Medical World News*, January 5, 1962; quoted in Kay 2000, p. 253.

6 Anon., “Science looks at life in 2057 A.D.,” *New York Times,* December 8, 1957, p. SM7.

7 Anon., “Science looks at life in 2057 A.D.,” *New York Times,* December 8, 1957, p. SM7.

8 See also Brandt (2009a), where the utopian views of Muller, Huxley, and Lederberg are analyzed in more detail in the context of literary traditions of science fiction.

9 J. B. S Haldane had developed similar ideas in his (1924) *Daedalus—Or Science and the Future*. In this utopian report of the future, Haldane developed imaginaries of *ectogenesis*: He wrote: “The small proportion of men and women who are selected as ancestors for the next generation are so undoubtedly superior to the average that the advance in each generation in any single respect, from the increased output of first-class music to the decreased convictions for theft, is very startling. Had it not been for ectogenesis there can be little doubt that civilization would have collapsed within a measurable time owing to the greater fertility of the less desirable members of the population in almost all countries” (Haldane 1924, pp. 66–67).

10 Briggs and King had just shown that the transplantation of cell nuclei from an advanced phase of cell differentiation could not induce normal embryonic development, and the results of the Fischberg and Gurdon group in Oxford, which pointed in a different direction, were probably not known to Muller at the time—this is evident from his text. Muller not only formulated a different view of the cell nucleus's potential than embryologists Briggs and King, but above all, he envisaged the possibilities as a thoroughly positive enrichment of society.

11 Around 1960, the genetic code was the focus of molecular biological research, but reliable experimental results of the so-called coding problem were still lacking (Kay 2000). Interestingly, Muller was skeptical about the possibilities of direct intervention since there would be no sufficient knowledge about the mode of action of genes in the “inordinately complex economy of the human organism” in the near future (Muller 1960, p.455). He viewed this as an argument for the further development of embryological methods (such as cell nuclear transfer), which, instead of genetic modification, aimed at the selection of entire genotypes, which he believed were “around the corner” (Muller 1960, p. 455).

12 Quotation from the cover of *Sexology. Authoritative Guide to Sex Education*, Vol. XXVIII, No. 11, June 1962.

13 H. J. Muller, “Improving Man’s Genes,” *Sexology*, June 1962, Muller manuscripts., Lilly Library, Indiana University, Bloomington, Indiana (hereafter, abbreviated to Muller Mss., series II, reprint No. 337/3 and 337/4.

14 “Germinal Choice, Script of Canadian TV program on germinal choice,” unpaginated, Muller Mss., series VII, Box 3.

15 Muller to Lederberg, Oct. 24, 1966, Joshua Lederberg papers, National Library of Medicine Archives, Bethesda, Maryland. Manuscript Collection MS C 552 (hereafter, Lederberg Papers), series III correspondence, Box 22, Folder 118.

16 Lederberg to Muller, November 2, 1966, Lederberg Papers, series III, correspondence, Box 22, Folder 118.

17 Lederberg to Aldous Huxley, January 23, 1963, Lederberg Papers, series III, correspondence 1961-1978, Box 19, Folder 201.

18 Joshua Lederberg, “Unpredictable Variety Still Rules Human Reproduction,” *Washington Post*, September 30, (1967), p. 17; Leon Kass, “Genetic Tampering”, Letter to the Editor of the *Washington Post*, October 30, 1967, two page newspaper clipping without pagination, Lederberg Papers, series IV, writings—Science and Man, Box 105, Folder 65.

19 “There may be a need one day, in the human situation at large, for one or more people especially constituted genetically to survive long periods outside a bathysphere at great marine depths or outside space capsules at great heights. If the greatest good for the greatest number were served, it would be justifiable not only to specialize people by cloning or constructive genetic engineering but also to bio-engineer or bio-design para-humans or ›modified‹ men as chimeras (part animal) or cyborg androids (part prostheses)” (Fletcher 1972, p. 83).

20 Letter from Philip Handler (National Academy of Sciences) to Lederberg, May 23, 1966, Lederberg Papers, Box 92, Folder 32.

21 National Academy of Sciences. Committee on Research in the Life Sciences: “Biology and the Future of Man,” February 1968, Lederberg Papers, Box 92, Folder 32 (hereafter abbreviated to: NAS report “Biology and the Future of Man”).

22 NAS report “Biology and the Future of Man,” p. 26.

23 NAS report “Biology and the Future of Man,” p. 27.

24 NAS report “Biology and the Future of Man,” p. 23.

25 NAS report “Biology and the Future of Man,” pp. 24–25.

26 See for example the very last sentences of the report: “Yet, man has overcome the limitations of his origin. Nature in him has reached beyond the hard regularities of physical phenomena and beyond the realization of organic configurations. Man, the creation of Nature, has transcended her. From a product of circumstances, he has risen to responsibility” (NAS report “Biology and the Future of Man,” p. 29).

27 “Stanford Biochemists Synthesize First Biologically Active DNA Virus,” Stanford *Medical Center Memo*, Vol. 9, No. 15, December 1967, quotation p. 1; Lederberg Papers, series XI subject files, Box 184, Folder 12.

28 Fred Harris, Chairman of the Subcommittee on Government Research, to Lederberg, February 26, 1968. See also “Introduction of Joint Resolution to Establish a Commission on Health, Science and Society” by Senator Walter Mondale, in Congressional Record Vol. 114, No. 19 (Senate - February 8, 1968), Lederberg Papers, series VII, writings, unpublished manuscripts, Box 88, Folder 44.

29 Flyer “From the desk of Senator Fred R. Harris, Oklahoma,” March 1, 1968, Lederberg Papers, series VII, writings, unpublished manuscripts, Box 88, Folder 44.

30 “Scientists Oppose ‘Biological Policy.’ Two Nobel prize-winning scientists from Stanford University joined yesterday to oppose Federal efforts at setting ethical policies in such controversial areas as organ transplants and genetic alterations,” Newspaper clipping, without date and title; see also: “Nobel Winners: ‘U.S shouldn’t set Biological Policy’,” *San Francisco Chronicle*, March 9, 1968, newspaper clipping without pagination, Lederberg Papers, series VII, writings, unpublished manuscripts, Box 88, Folder 44.

31 Harold M. Schmeck, Jr. “Scientist Doubts Genetic Abuse” [report on Kornberg’s statement], newspaper clipping, March 9, 1968 (without newspaper title). See also: Lederberg, “Replication of DNA Molecules Shouldn’t Be a Moral Issue,” *Washington Post*, January 6, 1968, newspaper clipping without pagination; Lederberg Papers, series VII, writings, unpublished manuscripts, Box 88, Folder 44.

32 Robert Reinhold, “The New Biology Taps Mysteries of Life,” *New York Times*, January 6, 1969, p. 144.

33 Robert Reinhold, “The New Biology Taps Mysteries of Life,” *New York Times*, January 6, 1969, p. 144.

34 Willard Gaylin, “The Frankenstein Myth Becomes a Reality—We Have the Awful Knowledge to Make Exact Copies of Human Beings,” *New York Times*, March 5, 1972, p. SM12ff.

35 Anon., “Wissenschaft: Mensch. Objekt für die Retorte,” *Der Spiegel*, Nr. 11, March 9, 1970, p. 169/170; quotation p. 170.

36 “Futurologie. Die Zukunft des Menschen wird geplant,” *Der Spiegel*, No. 53, Dec. 26, 1966, front cover.

37 Original: “Das ‚Experiment Menschheit,‘ die Selbstmanipulation des Menschen, hat begonnen, ja ist schon in vollem Gang und hat eine radikal neue Epoche der Menschheitsgeschichte eingeleitet. Die Forschung ist nämlich daran und strebt darauf hin, auch in das letzte Gefüge des Menschen und der Menschheit einzudringen, es umzugestalten und auf diesem Wege den uns heute bekannten Menschen, man möchte fast sagen, regelrecht abzuschaffen und ein andersartiges Wesen an seine Stelle zu setzen.”

38 Original: “das ungeheuerliche Problem der Selbstbedrohung der Menschheit durch die Naturwissenschaft ist hoch aktuell”

39 For more on the organism in biology in the interwar period see Baedke 2019; Erika Milam (2010) has shown how the idea of an “organismic biology” as a reaction against the physical and chemical approach of molecular biology took also shape in the United States in the 1960s, especially through the work of evolutionary biologist Ernst Mayr.

40 Original: “Mit der Eroberung des Raumes, der zielsicheren Entschleierung der Geheimnisse der lebendigen Substanz und des Vererbungsmechanismus haben wir uns auf einen Weg begeben, dessen Perspektive noch das späte 19., noch das frühe 20. Jahrhundert mit Entsetzen erfüllt hätte, wäre ihnen seine Phantastik auch nur einen Augenblick realisierbar vorgekommen. Denn die Supermen der Science Fiction haben nur wenig mit Nietzsches Übermenschen zu tun, der als ein Letztes, ein Ende und eine Erfüllung gedacht war, als die Krönung einer guten, nicht als das Gespenst einer schlechten Unendlichkeit. […] Eine prometheische Kultur hat kein Maß und kennt kein Tremendum. Sie steht unter dem Gesetz der Grenzenlosigkeit des Könnens und des Sieges über alle Widerstände, ein Prinzip, das seine faustischen Anfänge, wenn überhaupt, längst hinter sich gelassen hat.”

41 Original: “Die Biochemie hat in den Genen der Keimzelle den Lebensplan entdeckt. Er ist die in die Gene eingeschriebene, dort gespeicherte Vorschrift, das Programm der Entwicklung. Die Wissenschaft kennt bereits das Alphabet dieser Vorschrift. Man spricht vom „Archiv für die genetische Information“. Auf seine Kenntnis gründet man die sichere Aussicht, eines Tages die wissenschaftlich-technische Herstellbarkeit und Züchtung des Menschen in den Griff zu bekommen. Der Einbruch in die Genstruktur der menschlichen Keimzelle durch die Biochemie und die Atomzertrümmerung durch die Kernphysik liegen auf der selben Bahn des Sieges der Methode über die Wissenschaft.”

42 Martin Heidegger, TV interview with Richard Wisser, 1969; on the occasion of Heidegger’s eightieth birthday, ZDF broadcast a conversation between Wisser and the philosopher on September 24, 1969; https://www.youtube.com/watch?v=k7S0xER_cg0. Quotation at minute 12:43 (accessed January 5, 2025).

43 Original: “Wir würden die Entwürfe der Genetiker gerne als utopisch und kritiklos beiseite lege, wenn im Zeitalter des steigenden Tempos zwischen Utopie und Realisierung oft nicht nur wenige Jahre lägen. Wir müssen uns also der Weltaggression der modernen Genetik stellen, um die Stunde ihrer Realisierung nicht zu verschlafen.” On Stauder/Regau see also the “Literaturportal Bayern:” https://www.literaturportal-bayern.de/autorenlexikon?task=lpbauthor.default&pnd=107693801 (accessed January 5, 2025).

44 Original: “Wir werden sehen, daß avantgardistische Biologen […] die grotesken Phantasien des englischen Romanciers (that is: Aldous Huxley’s Brave New World, C.B.), nicht nur praktisch Schritt für Schritt einholen, sondern theoretisch weit überflügeln. Aber es gehört zu den Kennzeichen der Epoche, daß die Phantasien der Wissenschaftler die Phantasie der Romanautoren längst übertrifft. Nur bereiten uns die Erfindungen der Forscher weit mehr Ängste, als uns die Phantasien der Romanschreiber Vergnügen bereitet hatten” (Regau 1967 [1965], p. 189).

45 Anon., “Wissenschaft: Mensch. Objekt für die Retorte,” *Der Spiegel*, Nr. 11, March 9, 1970, p. 169-170; quotation p. 169.

46 Anon., “Wissenschaft: Mensch. Objekt für die Retorte,” *Der Spiegel*, Nr. 11, March 9, 1970, p. 169-170; quotation p. 170.

47 Original: “Dabei nimmt sich aber vor ihnen das unter den Auspizien der „Wissenschaft“ durchgeführte Massenexperiment des Nationalsozialismus, so unbegreiflich und grauenerregend es ist, fast wie ein provinzielles Ereignis aus. Was damals im Herzen Europas aufkam, hat jetzt große Teile der Welt ergriffen. Was damals einer Kooperation von Staat und Forschung entsprang, ist nunmehr in das Zentrum der Wissenschaft eingedrungen. […] In Gestalt des aufs feinste verteilten Terrors im Wirkungsbereich der modernen Großmacht, der Naturwissenschaft, droht diese „biologische Revolution“ nunmehr mit Hilfe einer „wissenschaftlich“ gesteuerten weltweiten Bewußtseinsindustrie, die eine Industrialisierung selbst der Entstehung des Menschens betreibt, unwiderstehlich zu werden.”

48 Victor K. McElheny, “Genetic Engineering Is a Rapidly Advancing Science,” *New York Times*, December 15, 1975, p. L 37.

49 See preceding section.

50 “Prospects for Designed Genetic Change. A Transcript Report from The National Advisory General Medical Sciences Council of the National Institute of General Medical Sciences, National Institutes of Health, Bethesda, Maryland, March 20, 1970,” Berg Papers, SC 358 ARCH 1989-062, Box14, Folder: “News Articles Prior to 1974” (hereafter: Prospects for Designed Genetic Change).

51 Prospects for Designed Genetic Change, quotations from preface.

52 Prospects for Designed Genetic Change, p. 1.

53 Gordon Tomkins, “Specific Problems in Higher Organisms,” and Vasken Aposhian,”Prospects for Therapeutic Change,” in Prospects for Designed Genetic Change, p. 17-31.

54 Prospects for Designed Genetic Change, p. 33.

55 Prospects for Designed Genetic Change, p. 33.

56 Prospects for Designed Genetic Change, p. 33.

57 Prospects for Designed Genetic Change, pp. 31–32.

58 “International Science Policy. A compilation of Papers Prepared for the 12th Meeting of the Panel on Science and Technology. Committee on Science and Astronautics. U.S. House of Representatives, May 1971,” Lederberg Papers, series V, writings – SAM Files, Box 110, Folder 31 (hereafter, International Science Policy).

59 James Watson, “Potential Consequences of Experimentation with Human Eggs,” in International Science Policy, p. 149-161, quotation on p. 159.

60 Jonathan Spivak, “Some Scientists Seek to Alter Human Genes. But Critics Claim Research Could Lead to the Breeding of Physical, Mental Giants,” *Wall Street Journal*, December 1971, newspaper clipping (without date and pagination), Berg Papers, SC 358 ARCH 1989-062, Box14, Folder “News Article Prior to 1974.”

61 Joshua Lederberg, “Genetic Engineering, or the Amelioration of Genetic Defect,” *The Pharos of Alpha Omega Alpha Honor Medical Society*, Vol. 34, No. 1, January 1971, pages 9-12. quotation on page 9; Lederberg Papers, Box 84, Folder 21. See also (Lederberg 1970).

62 “Peril seen in Studies on Microbe Genetics,” *Medical Tribune*, October 13, 1971, newspaper clipping, without author and pagination; Berg Papers, SC 358 ACCN 1989-062, Box 14, Folder “News Articles Prior to 1974.”

63 “Prospects for Designed Genetic Change, quotation p. 1

64 “Human Genetic Engineering. No Brave New World but Brand New Medical Potentials,” *Medical World News*, May 11, 1973, pp. 45–57, quotation p. 45; newspaper clipping, without author, Berg Papers, SC 358 ACCN 1989-062, Box 14, Folder “News Articles Prior to 1974.”

65 “Human Genetic Engineering. No Brave New World but Brand New Medical Potentials,” *Medical World News*, May 11, 1973, pp. 45–57, newspaper clipping, without author, Berg Papers, SC 358 ACCN 1989-062, Box 14, Folder: “News Articles Prior to 1974.”

66 Paul Berg, David Baltimore, Herbert W. Boyer, Stanley N. Cohen, Ronald W. Davis, David S. Hogness, Daniel Nathans, Richard Roblin, James D. Watson, Scherman Weissman, Norton D. Zinder.

67 The organizing committee consisted of molecular biologists from the United States as well as the United Kingdom and Switzerland: Paul Berg (chair), David Baltimore (MIT), Sydney Brenner (Medical Research Council Laboratory for Molecular Biology, Cambridge), Niels K. Jerne (Basel Institute for Immunology), Richard Roblin III (Harvard Medical School and Massachusetts General Hospital), Maxine Singer (Laboratory of Biochemistry, National Cancer Institute, NIH). However, in the report of the organizing committee to the National Academy of Sciences it is noted that Niels Jerne could not participate in the pre-conference activities and was unable to attend the conference (*Report of the Organizing Committee of the Asilomar Conference on Recombinant DNA Molecules*, Berg Papers, SC 358 ACCN 1989-062, Box 13, Folder: “photocopy of original final report.”

68 “Human Genetic Engineering. No Brave New World but Brand New Medical Potentials," *Medical World News*, May 11, 1973, pp. 45–57, quotation p. 45; newspaper clipping, without author, Berg Papers, SC 358 ACCN 1989-062, Box 14,Folder, "News Articles Prior to 1974.”

69 Kenneth Quade to Paul Berg, February 3, 1975, Berg Papers, SC 358 ACCN 1989-062, Box 13, Folder: “Correspondence, pre-Asilomar.”

70 Paul Berg’s handwritten note on the letter of Quade to Berg, February 3, 1975, Berg Papers, SC 358 ACCN 1989-062, Box 13, Folder ”Correspondence, pre-Asilomar.”

71 Anon., “Doomsday/ Cover Story. Tinkering with Life,” *Time*, April 18, 1977, p. 33; Berg Papers, SC 358 ARCH-1989-062, Box 25, Folder: “Newspaper clippings.”

72 Among the many international crises, a few are worth mentioning here. The beginning of the new decade was marked by political crises in the United States (such as Watergate) and, in Western countries, by a severe economic crisis in the wake of the first oil crisis. The Club of Rome’s 1972 report revealed the limits of growth and raised awareness about future ecological crises.

73 James Watson, “Recombinant DNA Research—Are We Proposing Regulations over Imaginary Dangers? Remarks prepared for a Hearing before the Attorney General of the State New York, October 21, 1976,” Berg Papers, SC 358, ACCN 1989-062,Box 15, Folder “Published Reactions to NIH Guidelines on RD research.”

74 “Biochemie: Der Mensch wird umgebaut,” *Der Spiegel*, No. 52, December 20, 1970 (cover page).

75 Anon., “Forschung: Embryologie: Eier im Glas,” *Der Spiegel*, No. 20, May 12, 1968, p. 170.

76 Anon., “Forschung: Biochemie: Senkrecht zur Hölle,” *Der Spiegel*, No. 52, December 20, 1970, p. 114.

77 Anon.,“Forschung/Genetik: Helden zu Hunderten,” *Der Spiegel*, No. 43, October 20, 1968; Anon., “Forschung: Biochemie: Senkrecht zur Hölle,” *Der Spiegel*, No. 52, December 20, 1970; Anon.,“Genetik: Eine Stadt voller Marilyn Monroes,” *Der Spiegel*, No. 39, September 23, 1973; Anon., “Genetik: Tausendmal schlimmer als Hitler,” *Der Spiegel*, No.12, March 26, 1978; Anon., “Ein Schritt in Richtung Homunkulus,” *Der Spiegel*, No. 31, July 30, 1978; Anon., “Genetik: Dutzendlinge, viele Dutzendlinge,” *Der Spiegel*, No. 3, January 11, 1981.

78 The German Bundestag finally passed a law on Regulation of Gene Technologies (Gentechnikgesetz) and the Embryo Protection Act in 1990.

79 A six-member citizens' commission convened by the mayor of Cambridge (Mass.) to advise the city council during a moratorium in 1976/1977 with respect to conducting genetic engineering experiments in laboratories at Harvard University (Waddell 1989).

80 Anon., “Genforschung in der politischen Diskussion. Synthetisiertes Leben,” *Bild der wissenschaft*, Nr. 12, 1977, 164-177.

81 Hans O. Hemmer interview with Erwin Chargaff: “Der Mensch macht sich zum Maßschneider des Schicksals,” August 1984 in Wengen/Switzerland, published in: Friedrich Ebert Stiftung, *Gewerkschaftliche Monatshefte* 1984, No. 10, p. 624-641, quotation, p. 631-632, translation C.B. (https://library.fes.de/gmh/jahresin.html, accessed 5 January 2025). Original: “Die Gefahr der Biotechnologie und der Genmanipulation liegt in der Brutalisierung der menschlichen Vorstellungskraft. Nach einiger Zeit wird alles möglich. Das Machbare wird nicht nur gemacht, es *muß* gemacht werden. Es wird ein Imperativ […]. Die Gentechnologie mache ich verantwortlich für die Exzesse der Reproduktionsforschung, die jetzt diese Scheußlichkeiten hervorbringt wie Retortenbabys, tiefgefrorene Embryos, die man eventuell sogar im Laden kaufen kann. Es ist die komplette Industrialisierung von Prozessen, die den Menschen bisher immer heilig waren. Wenn einmal das Ethos oder die moralischen Bedenken eines Volkes, einer Welt angefressen sind, dann wir von einem gewissen Moment an alles möglich […]. Die Auswüchse dieses Denkens münden dann in Vorstellungen vom „Klonieren“: der „Klon“ als vollständige Kopie des Vaters. Bei Mäusen hat man es theoretisch angeblich schon zustandegebracht. Ich habe meine Zweifel daran.”

82 Original: “Ich glaube, wir müssen bei der Risikodiskussion grundsätzlich verschiedene Dinge unterscheiden. Das eine ist die Diskussion von Risiken, die klar postuliert werden können und die man notfalls auch falsifizieren kann. Und das andere ist die Science fiction-artige Risikodiskussion.”

## References

[CR1] Abelson, Philip H.. 1971. Anxiety about genetic engineering. *Science* 173:285. 10.1126/science.173.3994.285 5559902 10.1126/science.173.3994.285

[CR2] Baedke, Jan. 2019. O organism, where art thou? Old and new challenges for organism-centered biology. *Journal of the History of Biology* 52:293–324. 10.1007/s10739-018-9549-4. 30465299 10.1007/s10739-018-9549-4

[CR3] Baitsch, Helmut. 1967a. Über die biologische Zukunft des Menschen. In *Menschliche Existenz und moderne Welt: Ein internationales Symposion zum Selbstverständnis des heutigen Menschen*, vol. 1, ed. Richard Schwarz, 656–669. Berlin: de Gruyter.

[CR4] Baitsch, Helmut. 1967b. Humangenetik. In *Der Mensch und seine Zukunft. Darmstädter Gespräch*, ed. Karl Schlechta, 56–64. Darmstadt: Neue Darmstädter Verlagsgesellschaft.

[CR5] Baitsch, Helmut. 1970. Das eugenische Konzept- einst und jetzt. In *Genetik und Gesellschaft: Marburger Forum Philippinum*, ed. Gerhard Wendt, 59–66. Stuttgart: Wissenschaftliche Verlagsgesellschaft.

[CR6] Beck, Ulrich. 1990 [1986]. *Risikogesellschaft: Auf dem Weg in eine andere Moderne*. Frankfurt am Main: Suhrkamp.

[CR7] Berg, Paul, David Baltimore, Herbert Boyer, Stanley Cohen, Ronald Davis, David Hogness, Daniel Nathans, et al. 1974. Potential biohazards of recombinant DNA molecules. *Science* 185:303. 10.1126/science.185.4148.303. 4600381

[CR8] Berg, Paul, David Baltimore, Sydney Brenner, Richard O. Roblin III., and Maxine F. Singer. 1975. Summary statement of the Asilomar conference on recombinant DNA molecules. *Proceedings of the National Academy of Sciences of the United States of America* 72:1981–1984. 10.1073/pnas.72.6.1981 806076 10.1073/pnas.72.6.1981 PMC432675

[CR9] Berg, Paul, and Maxine F. Singer. 1995. The recombinant DNA controversy: Twenty years later. *Proceedings of the National Academy of Sciences of the United States of America* 92:9011–9013. 10.1073/pnas.92.20.9011. 7568062 10.1073/pnas.92.20.9011 PMC40913

[CR10] Bösch, Frank. 2019. *Zeitenwende 1979: Als die Welt von heute begann*. München: Beck.

[CR11] Brandt, Christina. 2007. Wissenschaft – Literatur – Öffentlichkeit: Die Bedeutung der Science Fiction in den 1970er Jahren für die öffentliche Debatte zum Klonen. In *Wissenschaft und Öffentlichkeit als Ressourcen füreinander*, ed. Sybilla Nikolow and Arne Schirrmacher, 137–164. Frankfurt am Main: Campus.

[CR12] Brandt, Christina. 2009a. Die zwei (und mehr) Kulturen des Klons: Utopie und Fiktion im biowissenschaftlichen Diskurs der Nachkriegszeit. *NTM Zeitschrift**für Geschichte der Wissenschaften, Technik und Medizin* 17:243–275. 10.1007/s00048-009-0343-4 10.1007/s00048-009-0343-4 20027908

[CR13] Brandt, Christina. 2009b. In his Image: Klon-Experimente zwischen Biowissenschaft und Science-Fiction. In *Kulturgeschichte des Menschenversuchs im 20. Jahrhundert: Epistemologien – Settings – Repräsentationen*, eds. Birgit Griesecke, Markus Krause, Nicolas Pethes, and Katja Sabisch, 373–393. Frankfurt am Main: Suhrkamp.

[CR14] Briggs, Robert, and Thomas King. 1952. Transplantation of living nuclei from blastula cells into enucleated frogs’ eggs. *Proceedings of the National Academy of Sciences of the United States of America* 38:455–463. 10.1073/pnas.38.5.455. 16589125 10.1073/pnas.38.5.455 PMC1063586

[CR15] Brodde, Kirsten. 1992. *Wer hat Angst vor DNS? Die Karriere des Themas Gentechnik in der deutschen Tagespresse von 1973–1989*. Frankfurt am Main: Lang.

[CR16] Bundesminister für Forschung und Technologie, ed. 1984. *Ethische und rechtliche Probleme der Anwendung zellbiologischer und gentechnischer Methoden am Menschen. Dokumentation eines Fachgesprächs im Bundesministerium für Forschung und Technologie*. München: Schweitzer.

[CR17] Campos, Luis A. 2025. Invoking Asilomar: The historic meeting’s legacy resists simple lessons. *Science* 387:480–481. 10.1126/science.adv7574. 39883770 10.1126/science.adv7574

[CR18] Campos, Luis A., and Francesco Cassata. 2025. Introduction: Revis(it)ing Asilomar. *Journal of the History of Biology* 58:9–20. 10.1007/s10739-025-09817-8. 40261599 10.1007/s10739-025-09817-8

[CR19] Carlson, Elof Axel. 1987. Eugenics and basic genetics in H. J. Muller’s approach to human genetics. *History and Philosophy of the Life Sciences* 9:57–78. 3303104

[CR20] Carlson, Elof Axel. 1995. The parallel lives of H. J. Muller and J. B. S. Haldane: Geneticists, eugenists, and futurists. In *Haldanes´s Daedalus revisited*, ed. Krishna R. Dronamraju, 90–101. Oxford: Oxford University Press.

[CR21] Cassata, Francesco, and Soraya de Chadarevian. 2025. Asilomar across the Atlantic: EMBO, EMBL, and the politics of scientific expertise. *Journal of the History of Biology* 58:95–132. 10.1007/s10739-025-09808-9. 40038174 10.1007/s10739-025-09808-9 PMC12098474

[CR22] Catenhusen, Wolf-Michael., and Hanna Neumeister, eds. 1987. *Chancen und Risiken der Gentechnologie: Dokumentation des Berichts an den Deutschen Bundestag. Enquete-Kommission des Deutschen Bundestages*. München: Schweitzer.

[CR23] Chargaff, Erwin. 1976. On the dangers of genetic meddling. *Science* 192:938–940. 10.1126/science.11643312. 11643312

[CR24] Cobb, Matthew. 2022. *As gods: A moral history of the genetic age*. New York: Basic Books.

[CR25] Cobb, Matthew. 2025. The curious incident of Crick in the night-time and other Asilomar enigmas. *Journal of the History of Biology* 58:49–66. 10.1007/s10739-025-09805-y. 40009275 10.1007/s10739-025-09805-y PMC12098393

[CR26] Cohen, Stanley N., Annie C. Y. Chang, Herbert W. Boyer, and Robert Helling. 1973. Construction of biologically functional bacterial plasmids in vitro. *Proceedings of the National Academy of Sciences of the United States of America* 70:3240–3244. 10.1073/pnas.70.11.3240. 4594039 10.1073/pnas.70.11.3240 PMC427208

[CR27] Crowe, Nathan. 2014. Cancer, conflict, and the development of nuclear transplantation techniques. *Journal of the History of Biology* 47:63–105. 10.1007/s10739-013-9359-7. 23818038 10.1007/s10739-013-9359-7

[CR28] Crowe, Nathan. 2021. *Forgotten clones: The birth of cloning and the biological revolution*. Pittsburgh: University of Pittsburgh Press.

[CR29] Die Grünen im Bundestag, AK Frauenpolitik & Sozialwissenschaftliche Forschung und Praxis für Frauen e.V., eds. 1986. *Frauen gegen Gentechnik und Reproduktionstechnik. Dokumentation zum Kongreß vom 19. – 21. April 1985 in Bonn*. Köln: Volksblatt Verlag.

[CR30] De Chadarevian, Soraya. 2005. Asilomar – ein Moratorium und was daraus geworden ist. *Gegenworte* 16: 74–77.

[CR31] Dobzhansky, Theodosius. 1966 [1962]. *Mankind evolving: The evolution of the human species*. New Haven: Yale University Press.

[CR32] Doering-Manteuffel, Anselm, and Lutz Raphael. 2012. *Nach dem Boom: Perspektiven auf die Zeitgeschichte seit 1970*. Göttingen: Vandenhoeck.

[CR33] Ferguson, Niall, Charles S. Maier, Erez Manela, and Daniel J. Sargent, eds. 2011. *The shock of the global: The 1970s in perspective*. Cambridge, Mass.: Harvard University Press.

[CR34] Fletcher, Joseph. 1972. New beginnings in life: A theologian´s response. In *The new genetics and the future of man*, ed. Michael P. Hamilton, 78–89. Grand Rapids: Eerdmans.

[CR35] Flöhl, Rainer, ed. 1985. *Genforschung – Fluch oder Segen? Interdisziplinäre Stellungnahmen*. München: J. Schweitzer Verlag.

[CR36] Fredrickson, Donald. 1991. Asilomar and recombinant DNA: The end of the beginning. In *Biomedial Politics*, ed. Kathi E. Hanna, 258–298. Washington: National Academy Press.

[CR37] Frost, Robert, Julian Huxley, Devereux C. Joseph, Ashley Montagu, Herman J. Muller, Bertrand Russell. 1959. Proceedings of “The Future of Man,” September 29, 1959. A symposium sponsored by Joseph E. Seagram and Sons, Inc., on the dedication of its headquarters building in New York. New York, publisher unidentified.

[CR38] Goodell, Rae S. 1979. Public involvement in the DNA controversy: The case of Cambridge, Massachusetts. *Science, Technology, & Human Values* 4:36–43. 10.1177/016224397900400205. 10.1177/016224397900400205 11662583

[CR39] Gottweis, Herbert. 1998. *Governing molecules: The discursive politics of genetic engineering in Europe and the United States*. Cambridge, Mass: MIT Press.

[CR40] Gurdon, John. 1962. The developmental capacity of nuclei taken from intestinal epithelium cells of feeding tadpoles. *Journal of Embryology and Experimental Morphology* 10:622–640. 13951335

[CR41] Gurdon, John, Thomas Elsdale, and Michael Fischberg. 1958. Sexually mature individuals of *Xenopus laevis* from the transplantation of single somatic nuclei. *Nature* 182:64–65. 10.1038/182064a0. 13566187 10.1038/182064a0

[CR42] Haldane, John Burdon Sanderson. 1924. *Daedalus – or science and the future.* London: Kegan Paul, Trench, Truber.

[CR43] Hamilton, Michael Pollock, ed. 1972. *The new genetics and the future of man*. Grand Rapids: Eerdmans.

[CR44] Heidegger, Martin. 2013 [1967]. The provenance of art and the destination of thought. *Journal of the British Society for Phenomenology* 44:119–128.

[CR45] Heidegger, Martin. 2020 [1967]. Die Herkunft der Kunst und die Bestimmung des Denkens. In *Martin Heidegger Gesamtausgabe*. Vol. 80.2 part 2: *Vorträge 1935 bis 1967*, ed. Günther Neumann, 1327–1346. Frankfurt am Main: Klostermann.

[CR46] Herbig, Jost. 1978. *Die Gen-Ingenieure: Durch Revolutionierung der Natur zum neuen Menschen?* München: Hanser.

[CR47] Herwig, Eckart, and Sabine Hübner, eds. 1980. *Chancen und Gefahren der Genforschung. Protokolle und Materialien zur Anhörung des Bundesministers für Forschung und Technologie in Bonn, 19. bis 21. September 1979.* München: Oldenbourg (= Sozialwissenschaftliche Reihe des Battelle-Instituts e.V., 4).

[CR48] Jackson, David A., Robert H. Symons, and Paul Berg. 1972. Biochemical method for inserting new genetic information into DNA of Simian virus 40: Circular SV40 DNA molecules containing lambda phage genes and the galactose operon of *Escherichia coli*. *Proceedings of the National Academy of Sciences of the United States of America* 69:2904–2909. 10.1073/pnas.69.10.2904. 4342968 10.1073/pnas.69.10.2904 PMC389671

[CR49] Jasanoff, Sheila. 2005. *Designs on nature: Science and democracy in Europe and the United States*. Princeton: Princeton University Press.

[CR50] Jungk, Robert, and Hans Josef Mundt, eds. 1966. *Das umstrittene Experiment: Der Mensch*. München: Desch.

[CR51] Kass, Leon R. 1971. The new biology: What price relieving man’s estate? Efforts to eradicate human suffering raise difficult and profound questions of theory and practice. *Science* 174:779–787. 10.1126/science.174.4011.779. 5120517 10.1126/science.174.4011.779

[CR52] Kass, Leon R. 1972. New beginnings in life. In *The new genetics and the future of man*, ed. Michael P. Hamilton, 15–63. Grand Rapids: Eerdmans.

[CR53] Kaufmann, Richard. 1964. *Die Menschenmacher: Die Zukunft des Menschen in einer biologisch gesteuerten Welt*. Frankfurt am Main: Fischer.

[CR54] Kay, Lily. 2000. *Who wrote the book of life? A history of the genetic code*. Stanford: Stanford University Press.

[CR55] Kevles, Daniel J.. 1995. *In the name of eugenics: Genetics and the use of human heredity*. Cambridge, Mass.: Harvard University Press.

[CR56] King, Thomas, and Robert Briggs. 1956. Serial transplantation of embryonic nuclei. *Cold Spring Harbor Symposia on Quantitative Biology* 21:271–290. 13433597 10.1101/sqb.1956.021.01.022

[CR57] Klassen, Anna. 2026. *Vom Labor in den Bundestag: Die politische Regulierung von Gentechnologie in den 1970er und 1980er Jahren*. Stuttgart: Kohlhammer (in press).

[CR58] Krimsky, Sheldon. 1984. *Genetic alchemy: The social history of the recombinant DNA controversy*. Cambridge, Mass.: MIT Press.

[CR59] Küttemeyer, Wilhelm. 1970 [1969]. Wissenschaft, Methode und Mensch, von der Medizin aus gesehen. In *Menschenzüchtung: Das Problem der genetischen Manipulation des Menschen*, ed. Richard Wagner, 113–133. München: Beck.

[CR60] Lederberg, Joshua. 1963. Molecular biology, eugenics and euphenics. *Nature* 198:428–429. 10.1038/198428a0. 13929012 10.1038/198428a0

[CR61] Lederberg, Joshua. 1966a. Die biologische Zukunft des Menschen. In *Das umstrittene Experiment: Der Mensch*, ed. Robert Jungk and Hans-Josef. Mundt, 292–301. München: Desch.

[CR62] Lederberg, Joshua. 1966b. Experimental genetics and human evolution. *Bulletin of the Atomic Scientists* 22:4–11.

[CR63] Lederberg, Joshua. 1966c. Experimental genetics and human evolution. *The American Naturalist* 100:519–531. 10.1086/282446.

[CR64] Lederberg, Joshua. 1967. Experimentelle Genetik und menschliche Evolution. In *Menschliche Existenz und moderne Welt: Ein internationales Symposion zum Selbstverständnis des heutigen Menschen*, vol. 1, ed. Richard Schwarz, 670–688. Berlin: de Gruyter.

[CR65] Lederberg, Joshua. 1970. Genetic engineering and the amelioration of genetic defect. *BioSciences* 20:1307–1310. 10.1093/bioscience/20.24.1307.

[CR66] Luria, Salvador Edward. 1972. Slippery when wet: Being an essay on science, technology, and responsibility. *Proceedings of the American Philosophical Society* 116:351–356.

[CR67] Maienschein, Jane. 2003. *Whose view of life? Embryos, cloning, and stem cells*. Cambridge, Mass.: Harvard University Press.

[CR68] Meloni, Maurizio. 2016. *Political biology: Science and social values in human heredity from eugenics to epigenetics*. Basingstoke, Hampshire: Palgrave Macmillan.

[CR69] Milam, Erika Lorraine. 2010. The equally wonderful field: Ernst Mayr and organismic biology. *Historical Studies in the Natural Sciences* 40:279–317. 10.1525/hsns.2010.40.3.279. 20845573 10.1525/hsns.2010.40.3.279

[CR70] Muller, Hermann J.. 1935. *Out of the night: A biologist´s view of the future*. New York: Vanguard.

[CR71] Muller, Hermann J.. 1960. The guidance of human evolution. In *Evolution after Darwin.* Vol. 2*: The evolution of man: Man, culture and society*, ed. Sol Tax, 423–462. Chicago: University of Chicago Press.

[CR72] Muller, Hermann J.. 1961. Should we weaken or strengthen our genetic heritage? *Daedalus* 90:432–450.

[CR73] Muller, Hermann J.. 1963. Genetic progress by voluntarily conducted germinal choice. In *Man and his future: A Ciba foundation volume*, ed. Gordon Wolstenholme, 247- 262. Boston, Toronto: Little, Brown and Company.

[CR74] Muller, Hermann J.. 1965. Means and aims in human genetic betterment. In *The control of human heredity and evolution*, ed. T. M. Sonneborn, 100–122. New York: Macmillan.

[CR75] Muller, Hermann J.. 1966. Genetischer Fortschritt durch planmäßige Samenwahl. In *Das umstrittene Experiment: Der Mensch*, ed. Robert Jungk and Hans-Josef. Mundt, 277–291. München: Desch.

[CR76] Overhage, Paul. 1967. *Experiment Menschheit: Die Steuerung der menschlichen Evolution*. Frankfurt am Main: Knecht.

[CR77] Paul, Diane. 1984. Eugenics and the left. *Journal of the History of Ideas* 45:567–590. 10.2307/2709374. 11611620

[CR78] Paul, Diane. 1998. *The politics of heredity: Essays on eugenics, biomedicine, and the nature-nurture debate*. Albany: State University of New York Press.

[CR79] Petermann, Heike. 2009. Die biologische Zukunft der Menschheit: Der Kontext des CIBA Symposiums‚ Man and his Future‘ (1962) und seine Rezeption. In *Ursprünge, Arten und Folgen des Konstrukts „Bevölkerung“ vor, im und nach dem „Dritten Reich.“ Zur Geschichte der deutschen Bevölkerungswissenschaft,* eds. Rainer Mackensen, Jürgen Reulecke, and Josef Ehmer, 393–414. Wiesbaden: VS Verlag.

[CR80] Plessner, Helmuth. 2004 [1966]. Unmenschlichkeit. In *Mit anderen Augen: Aspekte einer philosophischen Anthropologie*, ed. Helmuth Plessner*,* 198–208. Stuttgart: Reclam.

[CR81] Portmann, Adolf. 1970 [1969]. Utopisches in der Lebensforschung. In *Menschenzüchtung: Das Problem der genetischen Manipulation des Menschen*, ed. Richard Wagner, 67–94. München: Beck.

[CR82] Radkau, Joachim. 1988. Hiroshima und Asilomar: Die Inszenierung des Diskurses über die Gentechnik vor dem Hintergrund der Kernenergie-Kontroverse. *Geschichte und Gesellschaft* 14:329–363. 11634990

[CR83] Rasmussen, Nicolas. 2014. *Gene jockeys. Life science and the rise of biotech enterprise*. Baltimore: Johns Hopkins University Press.

[CR84] Regau, Thomas. 1967. *Menschen nach Maß: Werkstoff Mensch im Griff einer seelenlosen Wissenschaft*. München: Bechtle.

[CR85] Reiß, Christian. 2023. Was ist Biologie, ca. 1956? Die Wissenschaft vom Leben in intellektuellen Diskursen im Deutschland und Österreich vor und nach 1945. In *Von einer Wissenschaft des Lebens zu den Lebenswissenschaften in Zentraleuropa*, eds. Mitchell G. Ash and Juliane Mikoletzky, 129–143. Wien: LIT Verlag.

[CR86] Salem, Samia. 2013. *Die öffentliche Wahrnehmung der Gentechnik in der Bundesrepublik Deutschland seit den 1960er Jahren*. Stuttgart: Franz Steiner Verlag.

[CR87] Scheffler, Robin Wolfe. 2025. Asilomar goes underground: The long legacy of recombinant DNA hazard debates for the greater Boston area biotechnology industry. *Journal of the History of Biology* 58:67–93. 10.1007/s10739-025-09806-x. 40053196 10.1007/s10739-025-09806-x PMC12098395

[CR88] Schlechta, Karl, ed. 1967. *Der Mensch und seine Zukunft: Darmstädter Gespräch*. Darmstadt: Neue Darmstädter Verlagsgesellschaft.

[CR89] Schwabe, Gerhard Helmut. 1970 [1969]. Wissenschaft, Methode und Mensch in biologischer Sicht. In *Menschenzüchtung: Das Problem der genetischen Manipulation des Menschen*, ed. Richard Wagner, 95–112. München: Beck.

[CR90] Schwerin, Alexander von, Anna Klassen, and Christina Brandt. 2023. *Gentechnik und die Max-Planck-Gesellschaft: Ein Streitfall zwischen Wissenschaft, Politik und Öffentlichkeit* (= Ergebnisse des Forschungsprogramms Geschichte der Max-Planck-Gesellschaft, ed. F. Schmaltz, J. Renn, C. Reinhardt, J. Kocka, Preprint 24). Berlin: Max-Planck-Gesellschaft, 2023; 10.17617/2.3552463

[CR91] Seefried, Elke. 2015. *Zukünfte: Aufstieg und Krise der Zukunftsforschung*. Berlin: de Gruyter.

[CR92] Sigmund, Georg. 1965. Umkonstruktion des Menschen? *Hochland. Zeitschrift Für Alle Gebiete des Wissens und der Schönen Künste* 57:476–482.

[CR93] Singer, Maxine, and Dieter Söll. 1973. Guidelines for DNA hybrid molecules (letter from the members of the Gordon Conference to the presidents of the National Academy of Sciences and the Institute of Medicine). *Science* 181:1114. 10.1126/science.181.4105.1114.a. 11663279 10.1126/science.181.4105.1114

[CR94] Sonneborn, Tracy M., ed. 1965. *The control of human heredity and evolution*. New York: Macmillan.

[CR95] Taylor, Gordon Rattray. 1969. *Die biologische Zeitbombe: Revolution der modernen Biologie*. Aus dem Englischen übersetzt von Gert Kreibich, Rudolf Süss, Walter Adolf. Frankfurt am Main: Fischer [English original: *Biological Time Bomb*. London: Thames & Hudson, 1968].

[CR96] Toffler, Alvin. 1971 [1970]. *Der Zukunftsschock*. Übersetzung aus dem Amerikanischen unter Mitwirkung des Verfassers. München: Scherz-Verlag [English original: *Future Shock*. London: Pan Books, 1970].

[CR97] Waddell, Craig. 1989. Reasonableness versus rationality in the construction and justification of science policy decisions: The case of the Cambridge Experimentation Review Board. *Science, Technology & Human Values* 14:6–29. 10.1177/016224398901400102. 10.1177/016224398901400102 11650830

[CR98] Wagner, Friedrich. 1964a. *Die Wissenschaft und die gefährdete Welt: Eine Wissenschaftssoziologie der Atomphysik*. München: C. H. Beck.

[CR99] Wagner, Friedrich. 1964b. Manipulation des menschlichen Keimplasmas als Ausweg aus den Zivilisationsproblemen? Genesis und Genetik. *Universitas* 19:1065–1076.

[CR100] Wagner, Friedrich, ed. 1970a [1969].* . Menschenzüchtung: Das Problem der genetischen Manipulation des Menschen*. München: Beck.

[CR101] Wagner, Friedrich. 1970b [1969]. Vorwort. In *Menschenzüchtung: Das Problem der genetischen Manipulation des Menschen*, ed. Friedrich Wagner, 7–11. München: Beck.

[CR102] Wagner, Friedrich. 1970c [1969]. Die Manipulierung des Menschen durch Genwissenschaft. Geschichte, Methoden, Ziele und Folgen. In *Menschenzüchtung: Das Problem der genetischen Manipulation des Menschen*, ed. Friedrich Wagner, 13–49. München: Beck

[CR103] Watson, James, and John Tooze. 1981. *The DNA story: A documentary history of gene cloning*. San Francisco: W. H. Freemann.

[CR104] Waugaman, Paul G. 1969. The case for a national commission on health science and society. *Public Administration Review* 29:290–294.

[CR105] Wendt, Gerhard, ed. 1970. *Genetik und Gesellschaft: Marburger Forum Philippinum*. Stuttgart: Wissenschaftliche Verlagsgesellschaft.

[CR106] Wieland, Thomas. 2012. Rote Gentechnik und Öffentlichkeit: Von der grundlegenden Skepsis zur differenzierten Akzeptanz. In *Biotechnologie-Kommunikation: Kontroversen, Analysen, Aktivitäten*, ed. Marc-Denis. Weitze, Alfred Pühler, Wolfgang M. Heckl, Bernd Müller-Röber, Ortwin Renn, Peter Weingart, and Günther. Wess, 69–112. Berlin, Heidelberg: Springer.

[CR107] Wieser, Wolfgang. 1963. Die genetische Utopie. *Merkur. Zeitschrift für Europäisches Denken* 17:321–337.

[CR108] Wieser, Wolfgang. 1966. Einleitung. Das umstrittene Experiment: Der Mensch – Grenzen und Möglichkeiten wissenschaftlicher Prognosen. In *Das umstrittene Experiment: Der Mensch*, eds. Robert Jungk and Hans-Josef Mundt, 9–26. München: Desch.

[CR109] Wright, Susan. 1994. *Molecular politics: Developing American and British regulatory policy for genetic engineering, 1972–1982*. Chicago: University of Chicago Press.

[CR110] Yi, Dogaab. 2015. *The recombinant university: Genetic engineering and the emergence of Stanford biotechnology*. Chicago: University of Chicago Press.

[CR111] Yi, Dogaab. 2025. Asilomar, gene cloning’s origins, and its commercial fate. *Journal of the History of Biology* 58:21–47. 10.1007/s10739-025-09803-0. 39966245 10.1007/s10739-025-09803-0 PMC12098428

